# Evolution of the Quorum network and the mobilome (plasmids and bacteriophages) in clinical strains of *Acinetobacter baumannii* during a decade

**DOI:** 10.1038/s41598-018-20847-7

**Published:** 2018-02-06

**Authors:** M. López, A. Rueda, J. P. Florido, L. Blasco, L. Fernández-García, R. Trastoy, F. Fernández-Cuenca, L. Martínez-Martínez, J. Vila, A. Pascual, G. Bou, M. Tomas

**Affiliations:** 1 Departamento de Microbiología, Instituto de Investigación Biomédica de A Coruña (INIBIC), Complexo Hospitalario Universitario de A Coruña (CHUAC), Sergas, Universidade da Coruña (UDC), Coruña, Spain; 2 Genomics and Bioinformatics Platform of Andalusia, GBPA, Seville, Spain; 3 Clinical Bioinformatics Research Area, Fundación Progreso y Salud (FPS), Hospital Virgen del Rocío, Seville, Spain; 4 Unidad de Enfermedades Infecciosas y Microbiología. Hospital Universitario Virgen Macarena. Departamento de Microbiología. Universidad de Sevilla. Instituto de Biomedicina de Sevilla (IBIS), Sevilla, Spain; 5 Departamento de Microbiología, Universidad de Córdoba, Córdoba, Spain; 6 Unidad de Gestión Clínica de Microbiología, Hospital Universitario Reina Sofia, Cordoba, Spain; 7 Instituto Maimónides de Investigación Biomédica de Córdoba (IMIBIC), Córdoba, Spain; 8 IS Global, Barcelona Centre for International Health Research (CRESIB), Hospital Clínic-Universitat de Barcelona, Barcelona, Spain

## Abstract

In this study, we compared eighteen clinical strains of *A*. *baumannii* belonging to the ST-2 clone and isolated from patients in the same intensive care unit (ICU) in 2000 (9 strains referred to collectively as Ab_GEIH-2000) and 2010 (9 strains referred to collectively as Ab_GEIH-2010), during the GEIH-REIPI project (Umbrella BioProject PRJNA422585). We observed two main molecular differences between the Ab_GEIH-2010 and the Ab_GEIH-2000 collections, acquired over the course of the decade long sampling interval and involving the mobilome: i) a plasmid harbouring genes for *bla*_OXA 24/40_ ß-lactamase and *abKA/abkB* proteins of a toxin-antitoxin system; and ii) two temperate bacteriophages, Ab105-1ϕ (63 proteins) and Ab105-2ϕ (93 proteins), containing important viral defence proteins. Moreover, all Ab_GEIH-2010 strains contained a Quorum functional network of Quorum Sensing (QS) and Quorum Quenching (QQ) mechanisms, including a new QQ enzyme, AidA, which acts as a bacterial defence mechanism against the exogenous 3-oxo-C12-HSL. Interestingly, the infective capacity of the bacteriophages isolated in this study (Ab105-1ϕ and Ab105-2ϕ) was higher in the Ab_GEIH-2010 strains (carrying a functional Quorum network) than in the Ab_GEIH-2000 strains (carrying a deficient Quorum network), in which the bacteriophages showed little or no infectivity. This is the first study about the evolution of the Quorum network and the mobilome in clinical strains of *Acinetobacter baumannii* during a decade.

## Introduction

*Acinetobacter baumannii* is a successful nosocomial pathogen, especially in Intensive Care Units (ICUs), where sporadic disease outbreaks occur^1,2^. The species is now endemic in some ICUs and is the second or third most common pathogen in nosocomial settings^3^. This is due to the ability of nosocomial pathogens to persist in the hospital environment for long periods of time, as well as to the continued increase in multidrug resistance and to virulence and/or pathogenicity, which are mainly acquired via the mobile genomic elements (bacteriophages, plasmids and transposons referred to as the “mobilome”) that are the main driving forces for the genome^4^. Proliferation of mobile elements stimulates chromosomal recombination and rearrangement, leading to genome plasticity and contributing to enhanced genetic variability and adaptation^5^.

Prophages (temperate bacteriophages) can introduce a plethora of genes that provide different functions to their hosts (e.g. motility, quorum sensing and stress tolerance)^6^. For instance, bacteriophages can protect their hosts against secondary infections through exclusion of superinfection as they prevent similar phage particles from attaching to the host^7,8^. Such lysogenic conversions can take the form of stable phenotypic changes or increased host plasticity, thereby improving bacterial survival under stress conditions via the Quorum Sensing (QS) network and the SOS response^6,9^.

On the other hand, Quorum Quenching (QQ) mechanisms can effectively interfere with any of the key processes in QS systems and this capacity could potentially be exploited to quench QS and prevent microbial infections^10^. Naturally occurring QQ mechanisms act by blocking key steps of the QS system, such as signal generation, signal accumulation and signal reception. Microorganisms exist in a multi-species, competitive environment and have developed many survival strategies to gain benefits and compete for space, nutrition and ecological niches. One of these, QS interruption, is straightforward because bacteria that produce QQ agents can inhibit the QS-regulated behaviour of competing species and therefore obtain benefits or avoid being killed by other bacteria or viruses, so that the functional network comprises both QS and QQ mechanisms.

An AHL acylase, AmiE, has recently been identified in *Acinetobacter* sp. strain Ooi24^11,12^. Our research group has recently described a new QQ enzyme (AidA) in a clinical isolate of *Acinetobacter baumannii* under 3-oxo-C12-HSL pressure^13,14^. Array studies revealed that only 13 proteins associated with 7 groups of families with the QQ phenotype were overexpressed in the presence of this molecule: the AidA enzyme (QQ mechanism), Glutathione-S-transferase (detoxification and DNA repair), RND transporter (efflux pump), Omp38 (OmpA), Enterocidin Ecn A/B family (stress response), Porin and 7 proteins involved in AHLs synthesis. The AidA enzyme was found to be associated with bacterial competition, as it is capable of hydrolyzing the signalling molecules used to mediate communication between species, including 3-oxo-C12-HSL^13^.

Information about bacteriophages and the network of QS/QQ mechanisms adds to a growing appreciation that plasmid carriage can have complex effects on the bacterial phenotype. For example, plasmids have been shown to alter biofilm formation^15,16^, cell hydrophobicity^17^, stress tolerance and motility^16^. Plasmid carriage can also alter biotic interactions with bacteriophages, limiting the coevolution of bacteria and bacteriophages and altering the longer-term evolutionary trajectory of bacterial populations^18^. The plasmid-harbouring bacteria have evolved lower resistance to bacteriophages, and plasmid-carrying bacteriophages have evolved lower infectivity than plasmid-free populations^18^.

In this study, we compared the genomes of eighteen clinical strains of *A*. *baumannii* belonging to the ST-2 clone and isolated from the same hospital and ICU (9 strains referred as Ab_GEIH-2000 and 9 strains referred as Ab_GEIH-2010) during the Spanish Multicentre Study (the GEIH-REIPI project). The aims of studying the collections of clinical *A*. *baumannii* strains were as follows: i) to investigate the molecular evolution of the strains by sequencing and carrying out genome analyses; ii) to develop linear sequences of the bacteriophages (Ab105-1φ and Ab105-2φ) from the representative strain of the Ab_GEIH-2010 collection (Ab105_GEIH-2010); iii) to characterize both bacteriophages (Ab105-1φ and Ab105-2φ) in the Ab_GEIH-2010 clinical strains; iv) to carry out functional analysis of the network of Quorum Sensing/Quorum Quenching systems in the isolates belonging to the Ab_GEIH-2000/2010 collections; and v) to determine the infective capacity of bacteriophages (Ab105-1φ and Ab105-2φ) in isolates from the Ab_GEIH-2000/2010 collections under stress conditions (3-oxo-C12-HSL) in relation to the QQ enzyme, AidA.

## Results

### Genomic and phenotypic features of the isolates of *A*.*baumannii* from Ab_GEIH-2000/2010 collections

Table 1 shows the results of whole-genome sequencing (WGS) studies undertaken as part of the GEIH-REIPI Spanish Multicentre *Acinetobacter baumannii* Study II 2000–2010, Umbrella BioProject PRJNA422585 (Bioproject Acc.Number in NCBI server). In total, 44.44% of the Ab_GEIH-2000 strains contained the AbATCC329/pMMCU3 plasmid harbouring *bla*_OXA 24/40_ ß-lactamase and *abKA/abkB* genes, in contrast to 100% of the Ab_GEIH-2010 isolates. Moreover, the MICs for the strains under study are shown in Table 2. The MICs of imipenem (64 mg/L) and meropenem (>128 mg/L) were the same for all Ab_GEIH-2010 strains, but not for the Ab_GEIH-2000 strains. Interestingly, the Ab_GEIH-2010 strains showed higher susceptibility than the Ab_GEIH-2000 strains to amikacin (resistance which is associated with aminoglycoside-modifying enzymes).

**Table 1:** Genome Sequences. Whole genome studies carried out as part of the GEIH-REIPI Spanish Multicentre *Acinetobacter baumannii* Study II 2000–2010 (Umbrella BioProject PRJNA422585).^a^CDSs: Coding Sequences for protein. ^b^AbATCC329/pMMCU3 plasmid harbouring *bl*a_OXA 24/40_ ß-lactamase gene and *abKA/abkB* genes from the TA system.

ST-2 Strain	GenBank Acc.Number^a^	Size (GC%)	ND50	Contigs	CDSs^a^	RNA genes	AbkAB toxin-antitoxin
Ab 155_GEIH-2000	LJHA00000000	3,991,758 (39.0%)	153263	64	3,759	63	−
Ab 158_GEIH-2000	MSMC00000000	3,860,705 (39.6%)	7301	885	3,507	22	+
Ab 161_GEIH-2000	MSMB00000000	3,848,902 (39.6%)	8375	820	3,483	24	+
Ab 166_GEIH-2000	MSMG00000000	3,572,409 (39.8%)	5197	1134	3,141	20	−
Ab 169_GEIH-2000	MSMF00000000	3,653,226 (39.5%)	6485	973	3,273	21	−
Ab 175_GEIH-2000	MSMI00000000	3,744,299 (39.7%)	7106	895	3,392	21	−
Ab 177_GEIH-2000	MSME00000000	3,896,859 (39.4%)	14751	511	3,617	27	−
Ab 183_GEIH-2000	MSMJ00000000	3,869,061 (39.5%)	8096	793	3,523	24	+
Ab 192_GEIH-2000	MSMH00000000	3,781,564 (39.4%)	13973	542	3,466	24	+
Ab 105_GEIH-2010	LJHB00000000	4,092,613 (39.0%)	112770	77	3,918	67	+
Ab 33_GEIH-2010	MSMK00000000	4,032,038 (39.3%)	16368	483	3,826	24	+
Ab 49_GEIH-2010	MSMM00000000	3,863,043 (39.4%)	10937	626	3,620	28	+
Ab 54_GEIH-2010	MSML00000000	3,972,430 (39.4%)	11936	655	3,707	29	+
Ab 76_GEIH-2010	MSLY00000000	3,862,509 (39.6%)	7456	888	3,523	24	+
Ab 103_GEIH-2010	MSLX00000000	3,883,445 (39.6%)	8217	850	3,559	24	+
Ab 104_GEIH-2010	MSMA00000000	3,960,610 (39.4%)	12369	650	3,690	25	+
Ab 121_GEIH-2010	MSLZ00000000	3,987,262 (39.3%)	16626	522	3,758	25	+
Ab 122_GEIH-2010	MSMD00000000	3,839,340 (39.6%)	8434	837	3,505	24	+

**Table 2:** Strains and susceptibility to several antimicrobials. CAZ: Ceftazidime; IMP: Imipenem; MER: Meropenem; SUL: Sulbactam; GEN: Gentamicin; TOB: Tobramycin; AMK: Amikacin; CIP: Ciprofloxacin; MIN: Minocicline; TIG: Tigecycline; COL: Colistin; DOR: Doripenem; DOX: Doxycycline; TET: Tetracycline; RIF: Rifampicin. Strains *E*. *coli* ATCC25922 and *P*. *aeruginosa* ATCC27853 strains were used as controls.

	ST-2 Strain	CAZ	IMP	MER	SUL	GEN	TOB	AMK	CIP	MIN	TIG	COL	DOR	DOX	TET	RIF
Ab_GEIH-2000	Ab 155_GEIH-2000	<0.12	0.12	0.06	128	0.25	0.5	1	0.5	4	16	0.25	<0.06	8	32	32
	Ab 158_GEIH-2000	>256	>128	>128	64	>128	8	64	0.25	8	32	0.25	>128	64	>128	2
	Ab 161_GEIH-2000	>256	>128	>128	32	>128	4	128	64	16	32	0.25	>128	>64	>128	4
	Ab 166_GEIH-2000	32	2	16	2	32	2	1	32	16	16	0.06	32	64	>128	4
	Ab 169_GEIH-2000	64	1	1	2	>128	4	512	128	8	16	0.12	>128	64	>128	2
	Ab 175_GEIH-2000	16	8	4	4	>128	4	128	128	0.5	16	0.25	>128	4	128	2
	Ab 177_GEIH-2000	128	4	8	4	>128	4	64	64	16	16	0.12	>128	64	>128	4
	Ab 183_GEIH-2000	>256	128	>128	64	>128	64	256	64	8	16	0.12	>128	64	>128	4
	Ab 192_GEIH-2000	>256	128	<128	32	>128	32	128	64	8	8	0.25	>128	64	>128	4
Ab_GEIH-2010	Ab 105_GEIH-2010	128	64	>128	128	>128	4	2	>128	8	16	0.12	>128	64	>128	4
	Ab 33_GEIH-2010	128	64	>128	32	>128	4	4	>128	16	32	0.25	>128	64	>128	4
	Ab 49_GEIH-2010	256	64	>128	32	8	64	32	64	1	0.5	<0.5	128	32	>128	2
	Ab 54_GEIH-2010	128	64	>128	128	>128	4	2	>128	8	8	0.12	>128	64	>128	4
	Ab 76_GEIH-2010	128	64	>128	64	>128	4	2	>128	8	16	0.25	>128	64	>128	4
	Ab 103_GEIH-2010	128	64	>128	64	>128	2	1	>128	8	16	0.25	>128	64	>128	64
	Ab 104_GEIH-2010	128	128	>128	128	>128	4	2	>128	16	8	0.25	>128	64	>128	64
	Ab 121_GEIH-2010	128	64	>128	128	>128	4	2	>128	16	16	0.12	>128	64	>128	4
	Ab 122_GEIH-2010	128	64	>128	128	>128	8	2	>128	8	8	0.25	>128	64	>128	4

**Figure 1:**
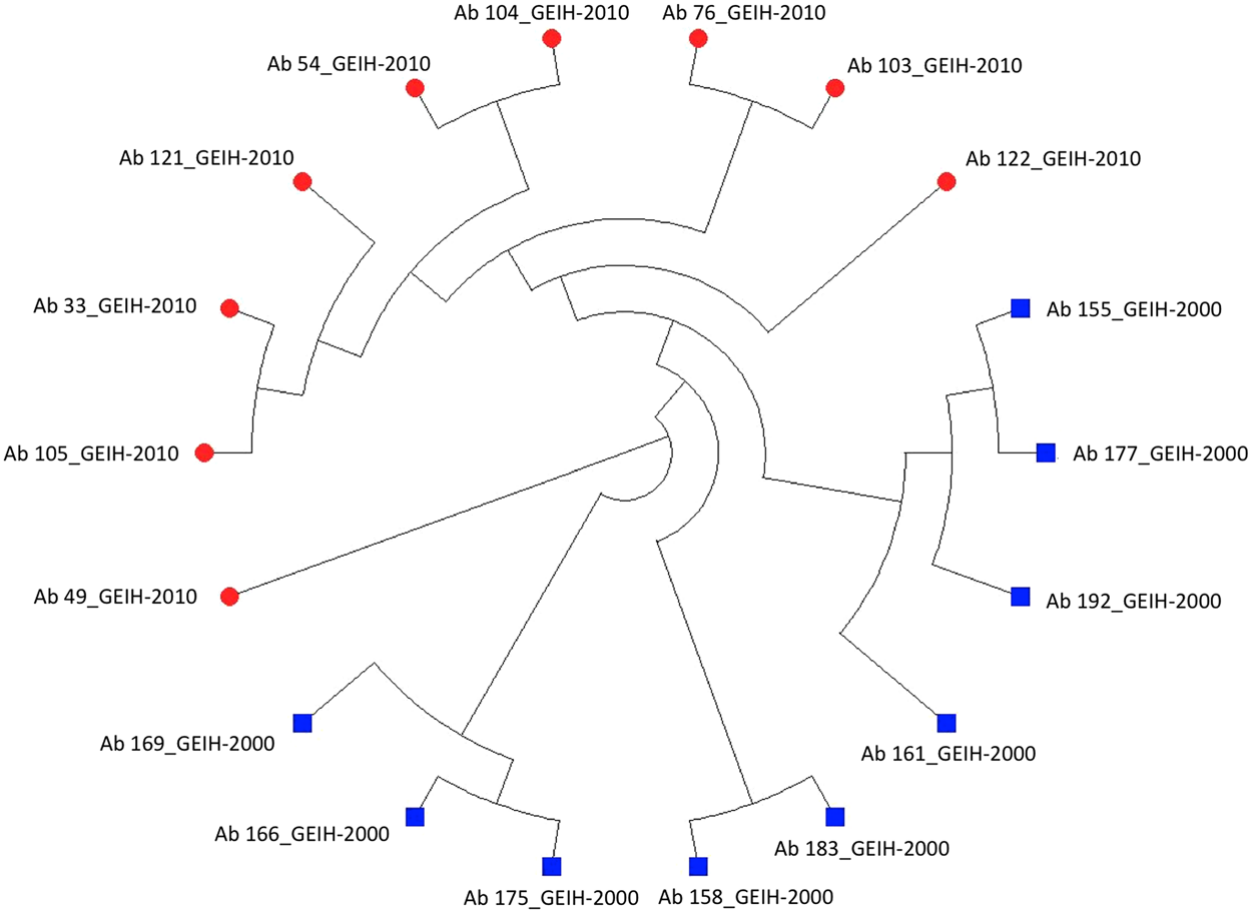
Phylogenetic tree with multiple alignment of Ab_GEIH-2000 and Ab_GEIH-2010 collections. The strains from Ab_GEIH-2000 are showed in blue versus isolates of Ab_GEIH-2010 in red colour.

The phylogenetic tree from both collections (Ab_GEIH-2000/2010) is shown in the Fig. 1. We observed the genomic similarity between strains of Ab_GEIH-2000 versus the genomic similarity of Ab_GEIH-2010 isolates.

**Figure 2:**
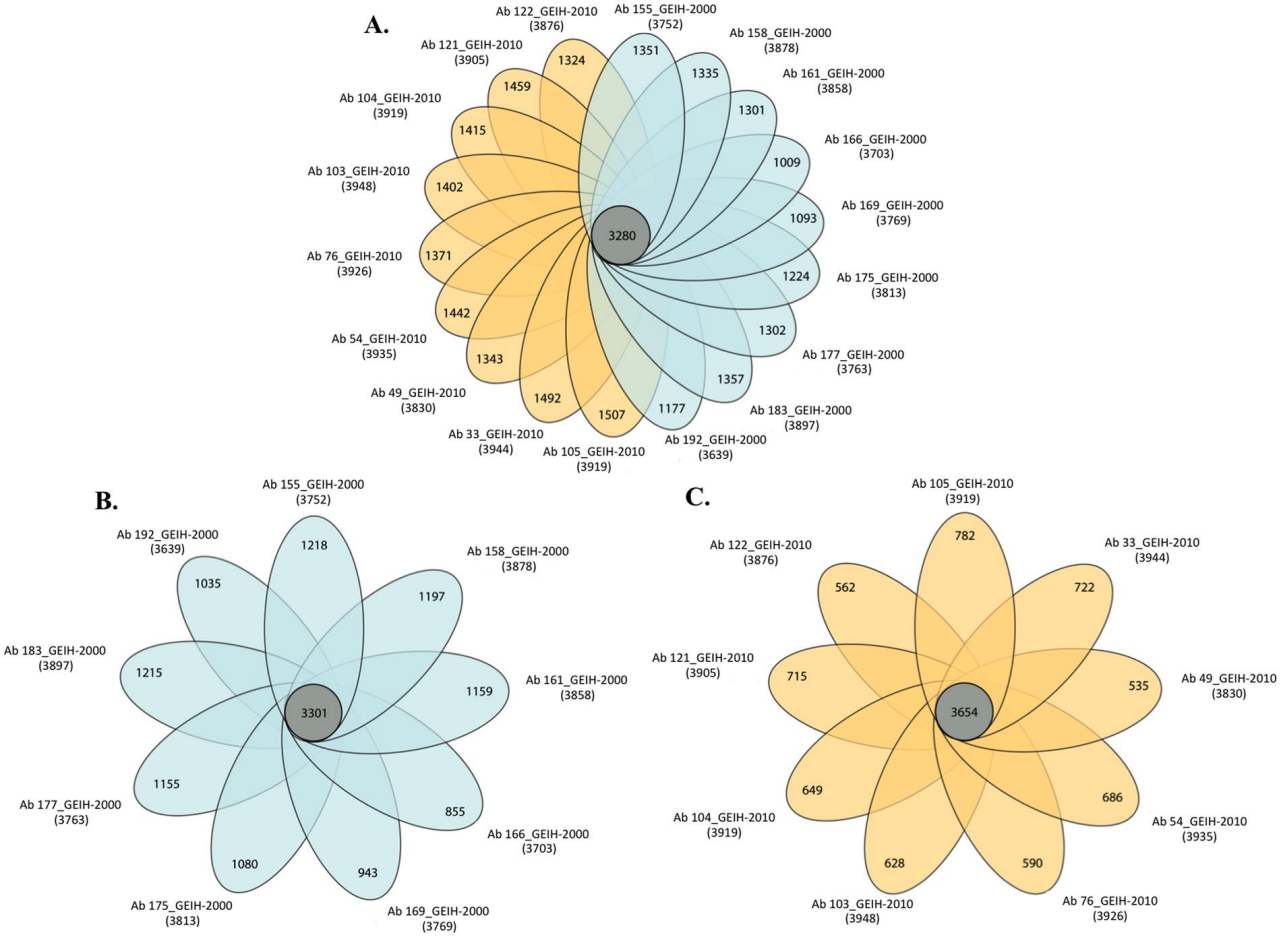
(**A**) Genomic comparison of the Ab_GEIH-2000 and Ab_GEIH-2010 collections of strains. The strains are represented by different coloured ovals depending on whether they are included in the Ab_GEIH-2000 (blue) or Ab_GEIH-2010 (yellow) collections. The number shown in the non-overlapping portions of each oval is the number of unique coding DNA sequences (CDSs) in each strain (accessory genome). The total number of CDSs within each genome is shown in brackets below the strain name, and the size of the core genome (orthologous CDSs shared by all strains) is shown in the centre of the figure. The figure was constructed using Adobe Illustrator. (**B**) Genomic comparison of Ab_GEIH-2000 strains. The core genome (centre), the accessory genome of each strain (blue oval). The total number of CDSs within each genome is shown in brackets below the strain name. (**C**) Genomic comparison of the strains in the Ab_GEIH-2010 collection. The core genome (centre), the accessory genome of each isolate (yellow oval). The total number of CDSs within each genome is shown in brackets below the strain name.

Moreover, comparison of the chromosomal sequences (Fig. 2) of the Ab_ GEIH-2000 and Ab_GEIH-2010 collections of strains revealed the following: i) the number of similar proteins (core-genome) was higher in the Ab_GEIH-2010 strains than in the Ab_GEIH-2000 strains (3654 proteins relative to 3301 proteins); ii) of these proteins (core-genome), 239 were located in bacteriophages in the Ab_GEIH-2010 strains, compared with 21 in the Ab_GEIH-2000 strains; and iii) of the 51 proteins present only in the Ab_GEIH-2010 strains (accessory genome), 40 proteins (79%) were located in the bacteriophages. Finally, comparison of the plasmidic sequences indicated the presence of 17 proteins only in the Ab_GEIH-2010 isolates (AbATCC329/pMMCU3 plasmid harbouring genes encoding OXA 24/40 ß-lactamase and AbKA/AbkB proteins)^19^.

**Figure 3:**
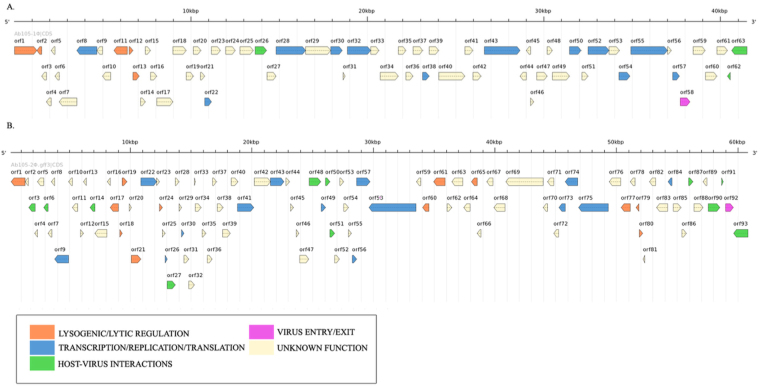
Genome annotation representation for bacteriophages Ab 105-1ϕ (**A**) and Ab 105-2ϕ (**B**). The genes were predicted using GenemarkS software (trained with existing annotation of *Acinetobacter baumannii* MDR-TJ). The resulting genes were annotated by integrating the output of Blast2Go, RAST, PHAST or PHASTER, or were manually annotated using BLAST software. This information was used to group the genes in different categories. The plot was constructed using genome tools; each box represents a predicted ORF and the arrow indicates the direction of the gene.

The genome of strain Ab105_GEIH-2010 (representative of the Ab_GEIH-2010 group of strains) included two temperate bacteriophages: Ab105-1ϕ (size 41,496 bp; 63 ORFs) and Ab105-2ϕ (size 61,304 bp; 93 ORFs). The sequences were deposited in GenBank under nucleotide sequence numbers KT588074 and KT588075, respectively. The phages showed the best BLAST matches in *Siphoviridae* bacteriophage family YMC/09/02/B1251 ABA BP (Bphi-B1251 [NC_019541.1]). All open reading frames (ORFs) comprising the two bacteriophage genomes, as determined by bioinformatic analysis and PCR techniques, are shown in Tables S2 and S3 (Supplementary files). Completely aligned phage genomes (ORFs) are shown in Fig. 3. Interestingly, the genome of the Ab155_GEIH-2000 strain (representative of Ab_GEIH-2000 strains) included phage proteins but no complete temperate bacteriophages.

**Figure 4:**
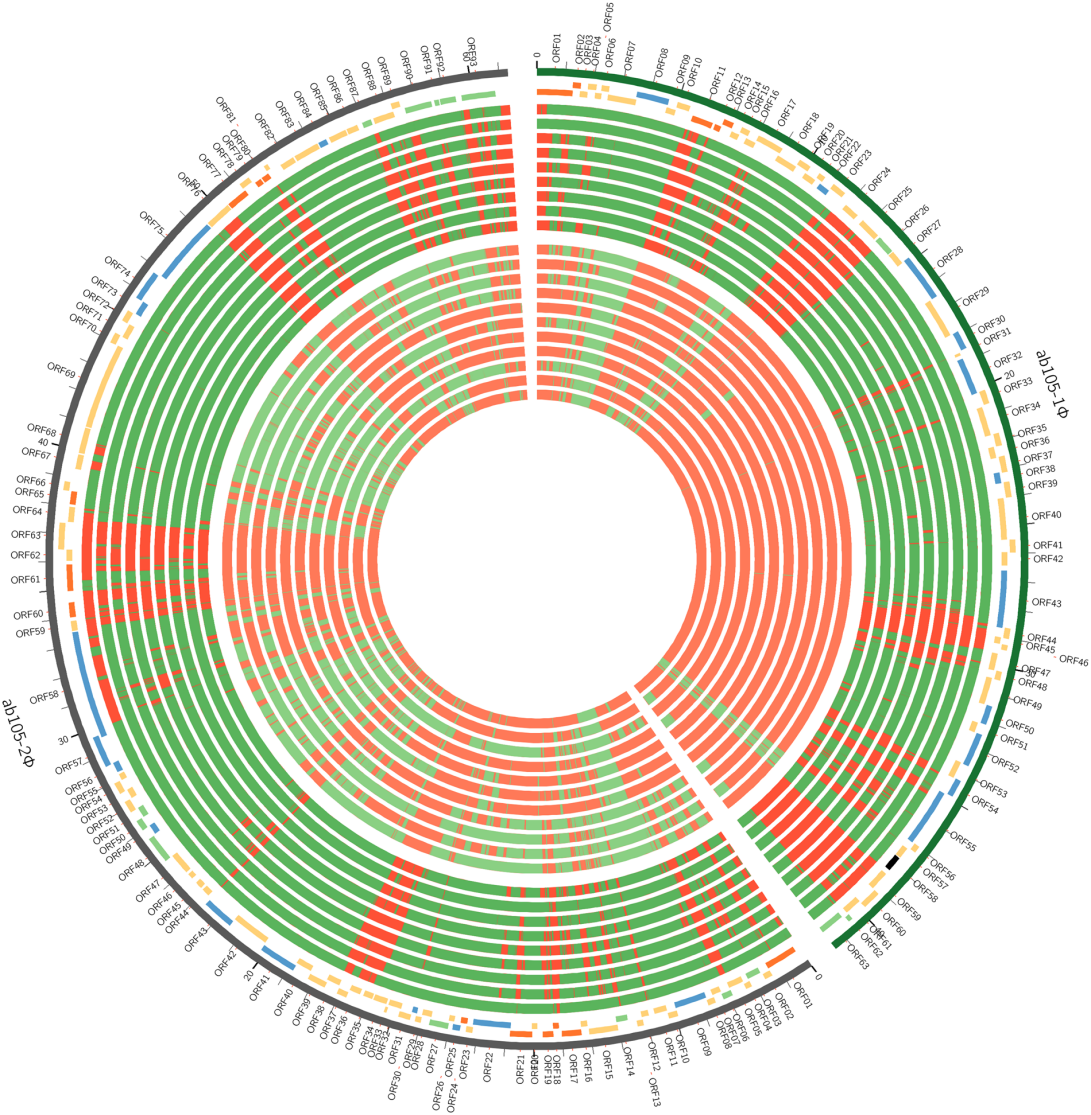
Genome of bacteriophages Ab 105-1ϕ and Ab 105-2ϕ in Ab_GEIH-2000/2010 strains. The circos plot represents the genome of bacteriophages Ab 105-1ϕ and Ab 105-2ϕ, as follows, from the outer to the inner track: (1) names of the predicted ORFs; (2) genomes of both bacteriophages; (3) track of genes coloured as before; (4) similarity between the genomes of the two bacteriophages and the sequence of the other strains isolated in 2010. Red-coloured areas indicate no similarity in that region, i.e. this region is not present in the other genome or it was not detected with sufficient confidence. Green-coloured areas indicate a high level of confidence regarding the presence of the region; and (5) Equivalent plot for the strains isolated in 2000. In order to create the similarity tracks, we aligned the reads from each against the whole assembly, by using BWA software package. We used *samtools* to select those that map with high confidence in the phage regions and *bedtools* to measure the coverage. Those regions with coverage of more than 25× (expected around 150×) were flagged as very similar.

Finally, we observed the presence of Ab105-1ϕ and Ab105-2ϕ bacteriophages in all Ab_GEIH-2010 strains but not in the Ab_GEIH-2000 strains (Fig. 4).

### Response of *A*. *baumannii* Ab105_GEIH-2010 strain (carrying of Ab105-1ϕ and Ab105-2ϕ bacteriophages) under the SOS response (MMC)

The SOS response (MMC) produced differences in microarray expression of the genes from the two bacteriophages of the Ab105_GEIH-2010. Under stress conditions by SOS response we observed an overexpression of 5% and 30% of genes by Ab105-1ϕ and Ab105-2ϕ bacteriophages respectively (Umbrella BioProject PRJNA422585). Only three ORFs were expressed in bacteriophage Ab105-1ϕ, while 28 ORFs were expressed in bacteriophage Ab105-2ϕ (30.10%) (Table 3). Moreover, up to 40 minutes after the addition of MMC (to induce the SOS response), overexpression of ORF27 (methyltransferase-SAM or AdoMet-MTase) with a relative expression (RE) of 200 times and ORF06 (MazG-like protein) with an RE of 81.3 times that of Ab105-2ϕ was observed by qRT-PCR. After 40 minutes, the RE of ORF 93 (DNA polymerase UmuC) increased by 10 times (phage Ab105-2ϕ).

**Table 3:** Microarrays studies. Expression of the Ab105_GEIH-2010 genome (determined by microarray analysis under the SOS response) and lytic/metabolic expression of Ab 105-1ϕ and Ab 105-2ϕ prophage genes. ^a^Proteins associated with viral defence against bacterial attack.

ORF	Description	General Function	Expression Level
**Ab 105-1ϕ prophage**			
ORF08	ATPaseAAA(domain protein)/Rec A-like family ATPases	Viral replication	10.7
ORF09	HP/UF	—	4.5
ORF10	HP/UF	—	3.4
**Ab 105-2ϕ prophage**			
ORF01	Integrase	Lysogenic state	3.26
ORF02	HP/UF	—	5.60
**ORF03**	**SAM or AdoMet-MTase**	**Viral defense** ^**a**^	**7**.**09**
ORF04	HP/UF	—	6.83
ORF05	Phage protein	—	7.90
**ORF06**	**MazG-like protein**	**Viral defense** ^**a**^	**10**.**96**
ORF07	HP/UF	—	10.39
ORF08	HP/UF	—	13.36
ORF09	ATPaseAAA(domain protein)/Rec A-like family ATPases	Viral replication	12.40
ORF11	HP/UF	—	16.75
ORF18	XRE family transcriptional regulator (repressor)	Lytic/ lysogenic regulation	4.35
ORF19	Transcriptional regulator (Lambda repressor-like)	Lytic/ lysogenic regulation	10.36
ORF20	HP/UF	—	9.17
ORF21	Phage replication, protein O	Viral replication	5.15
ORF22	Replicative DNA helicase	Viral replication	3.94
ORF23	HP/UF	—	4.59
ORF24	Integrase	Lysogenic state	5.12
ORF25	MarR family-transcriptional regulator	Lytic/ lysogenic regulation	4.20
ORF26	HP/UF	—	4.09
**ORF27**	**SAM or AdoMet-MTase**	**Viral defence** ^**a**^	**4**.**00**
ORF28	HP/UF	—	3.10
ORF29	HP/UF	—	3.22
ORF30	DnaJ-class molecular chaperone with C-terminal Zn finger domain, putative	DNA metabolism/replication	3.83
ORF32	HP/UF	—	2.54
ORF33	HP/UF	—	2.16
ORF58	HP/UF	—	3.07
ORF66	Rha transcriptional regulator	Lytic/ Lysogenic regulation	2.04
**ORF80**	**DNA polymerase UmuC** (**DNA-repair protein**, **UmuC-like**, **N-terminal**)	**Viral defence** ^**a**^	**2**.**73**

On the other hand, in the arrays results from the Ab105_GEIH-2010, there were other overexpressed bacterial genes involved in the SOS response under MMC (Umbrella BioProject PRJNA422585). Among them, we highlight several genes related to DNA repair with high overexpression (>10 fold): i) Glutathione S-transferase ii) Deoxyuridine 5 -triphosphate nucleotidohydrolase (dUTPase); iii) ParB-like protein; and iv) Thymidylate synthase.

**Figure 5:**
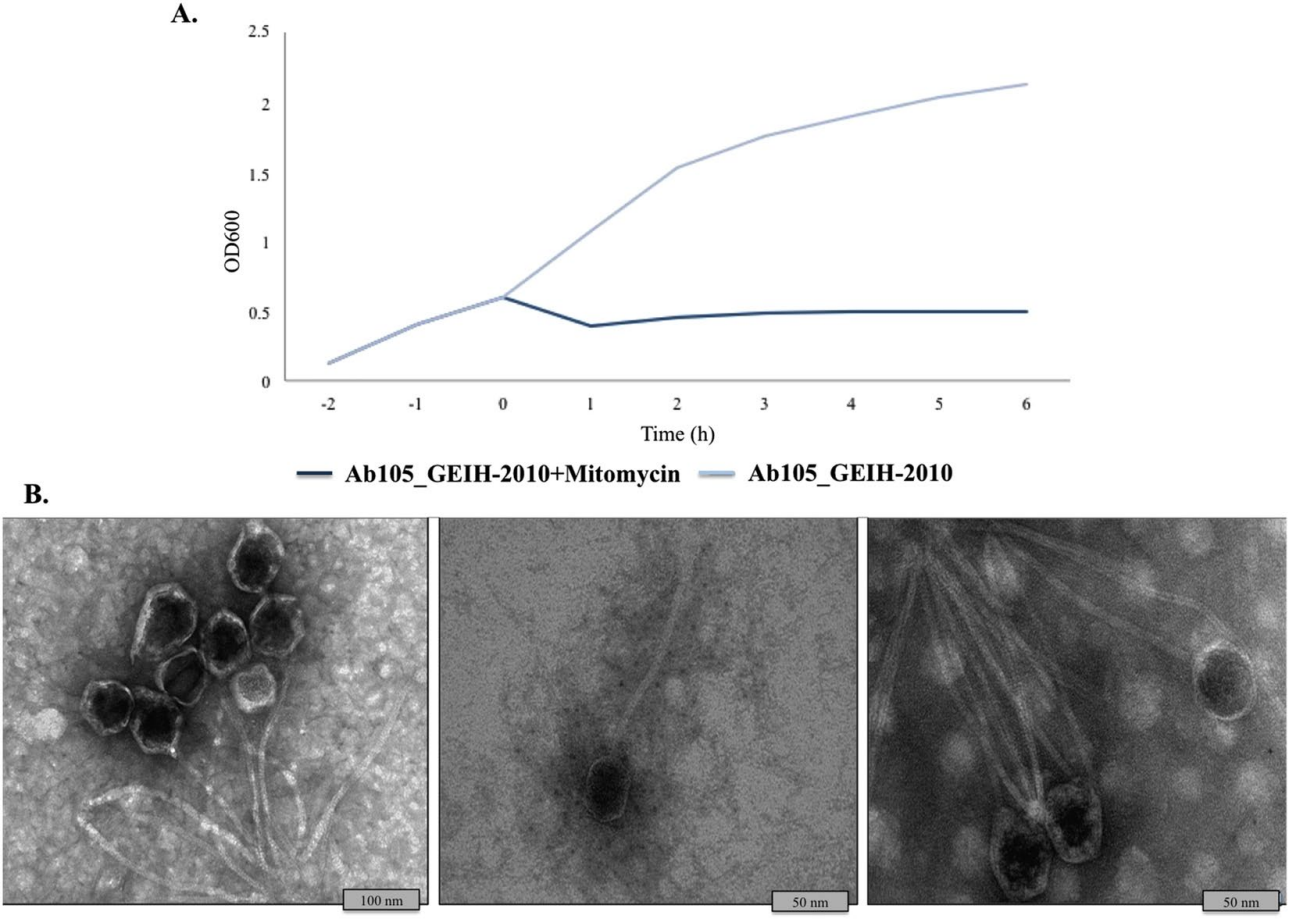
Growth curves. (**A**) Ab_GEIH-2010 strains under the SOS response (induced by MMC). (**B**) Bacteriophages examined by TEM microscopy.

Finally, the bacterial response after incubation with MMC is shown in Fig. 5. Bacterial lysis occurred in the presence of MMC but not in the absence of this compound. Finally, Fig. 5 also shows the characteristic morphology of the phages in the *Siphoviridae* family, with a long tail and an icosahedral capsid of diameter~ 60 nm (http://viralzone.expasy.org/).

### Responses of Ab_GEIH-2000/2010 collections under stress conditions (3-oxo-C12-HSL and H_2_O_2_)

The results of expression of the *abaI* (QS) and the *aidA* (QQ) genes in all strains from the Ab_GEIH-2000 and Ab_GEIH-2010 collections under stress conditions are shown in Table 4. Activation of the QS system by the ROS response (presence of H_2_O_2_) produced overexpression of the *abaI* gene in all of the Ab_GEIH-2010 strains. In the presence of the 3-oxo-C12-HSL molecule (used to induce inhibition of the QS system), all of the Ab_GEIH-2010 strains overexpressed the *aidA* gene, while only 55.5% of the Ab_GEIH-2000 strains overexpressed this QQ enzyme and 44.4% of these strains overexpressed the *abaI* gene. Therefore, the Ab_GEIH-2010 strains showed QS-deficient cells relative to the Ab_GEIH-2010 strains, which had a functional QS system.

**Table 4:** Relative expression (RE) of the Quorum sensing genes (*abaI* and *aidA*). **Mutations or deletions in the sequence of *abaR* gene. In bold type, those isolates with no or low expression of *aidA* gene; ^a^NCBI GenBank Database; ^b^Inhibition of the Quorum Sensing system by Quorum Quenching activity (induced by the presence of 3-oxo-C12-HSL); ^c^Activation of the Quorum Sensing system via the ROS response (induced by the presence of H_2_O_2_). NA, Not Applicable.

PROJECT	Strain of *A*. *baumannii* clone ST-2	*abaR*	Acc. Number. Genbank^a^		3-oxo-C12-HSL^b^		H_2_O_2_^c^		Bacterial sensitivity to the Bacteriophages Ab105-1ϕ and Ab105-2ϕ
			*abaI*	*aidA*	*abaI*	*aidA*	*abaI*	*aidA*	
Ab_GEIH-2000	Ab 155_GEIH-2000	ODA52665.1	ODA52667.1	ODA50170.1	1.20	**1**.**50**	**3**.**00**	0.50	−
	Ab 158_GEIH-2000	OLV45102.1	OLV48145.1	OLV47950.1	**3**.**63**	1.09	**2**.**10**	0.67	−
	Ab 161_GEIH-2000	OLV47680.1**	OLV49329.1	OLV44152.1	0.69	**1**.**75**	**2**.**25**	0.34	−
	Ab 166_GEIH-2000	−	−	−	NA	NA	NA	NA	**+**
	Ab 169_GEIH-2000	OLV61375.1**	OLV61377.1	OLV61862.1	0.50	**2**.**11**	**1**.**97**	1.00	−
	Ab 175_GEIH-2000	OLV76671.1**	OLV72445.1	−	**1**.**55**	NA	**2**.**89**	NA	−
	Ab 177_GEIH-2000	OLV61352.1	OLV61354.1	OLV60446.1	1.20	**2**.**39**	**1**.**60**	0.78	**+**
	Ab 183_GEIH-2000	OLV73449.1	OLV79706.1	OLV77156.1	**4**.**45**	0.34	**1**.**65**	0.67	−
	Ab 192_GEIH-2000	OLV66603.1	OLV66482.1	−	**6**.**19**	NA	**1**.**80**	NA	−
Ab_GEIH-2010	Ab 105_GEIH-2010	ODA55972.1	ODA55974.1	ODA53988.1	0.92	**3**.**94**	**1**.**55**	0.36	**+**
	Ab 33_GEIH-2010	OLV79037.1	OLV79035.1	OLV80563.1	1.10	**2**.**50**	**1**.**50**	0.50	**+**
	Ab 49_GEIH-2010	OLV89715.1	OLV84391.1	OLV86104.1	1.23	**6**.**49**	**2**.**10**	0.14	**+**
	Ab 54_GEIH-2010	OLV83069.1	OLV83067.1	OLV85628.1	1.09	**2**.**50**	**5**.**16**	0.20	**+**
	Ab 76_GEIH-2010	OLV40014.1	OLV29965.1	OLV34275.1	0.64	**1**.**80**	**2**.**20**	0.25	**+**
	Ab 103_GEIH-2010	OLV41119.1	OLV35969.1	OLV33173.1	1.44	**1**.**77**	**1**.**80**	0.10	**+**
	Ab 104_GEIH-2010	OLV48295.1	OLV40038.1	OVL46713.1	1.14	**2**.**62**	**2**.**14**	0.23	**+**
	Ab 121_GEIH-2010	OLV37874.1	OLV38310.1	OVL36028.1	0.80	**1**.**70**	**3**.**14**	0.20	**+**
	Ab 122_GEIH-2010	OLV58946.1	OLV53842.1	OVL52031.1	0.40	**3**.**73**	**1**.**75**	0.33	**+**

**Figure 6:**
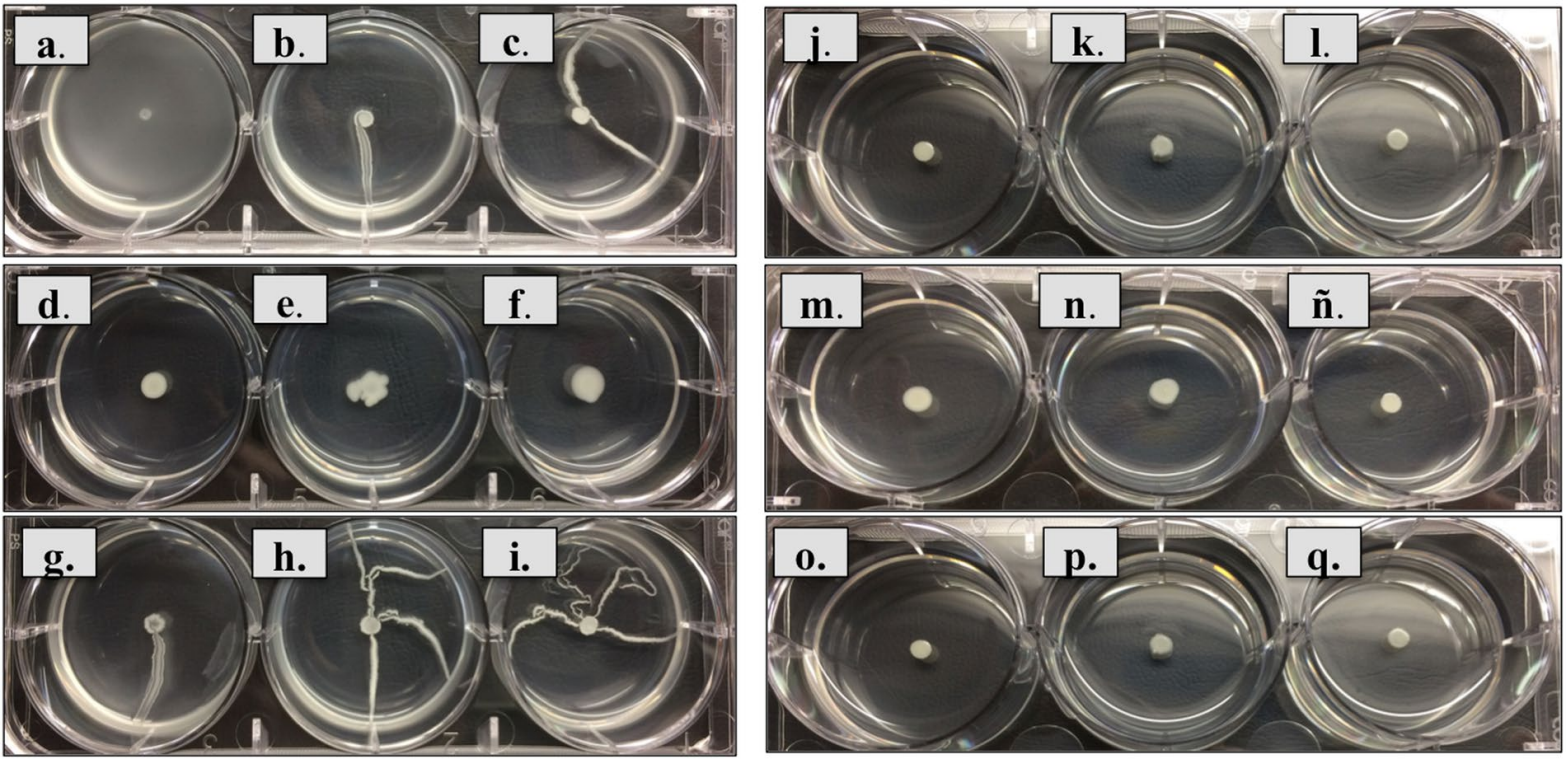
Motility of the Ab_GEIH-2000 and Ab_GEIH-2010 strains. (**a**) Ab155_ GEIH-2000; (**b**) Ab158_GEIH-2000; (**c**) Ab161_GEIH-2000; (**d**) Ab166_GEIH-2010; (**e**) Ab169_GEIH-2000; (**f**) Ab175_GEIH-2000; (**g**) Ab177_GEIH-2000; (**h**) Ab183_GEIH-2000; (**i**) Ab192_GEIH-2000; (**j**) Ab33_GEIH-2010; (**k**) Ab49_GEIH-2010; (**l**) Ab54_ GEIH-2010; (**m**) Ab76_GEIH-2010; (**n**) Ab103_GEIH-2010; (**ñ**) Ab104_GEIH-2010; (**o**) Ab105_GEIH-2010; (**p**) Ab121_GEIH-2010 and (**q**) Ab122_GEIH-2010.

Finally, the absence of surface motility profile was also homogeneous (QQ phenotype) in the Ab_GEIH-2010 strains relative to the heterogeneity associated with the presence of surface motility in some isolates of the Ab_GEIH-2000 collection (Fig. 6).

### Impact of the Ab105-1ϕ and Ab105-2ϕ bacteriophages: infective capacity

**Figure 7:**
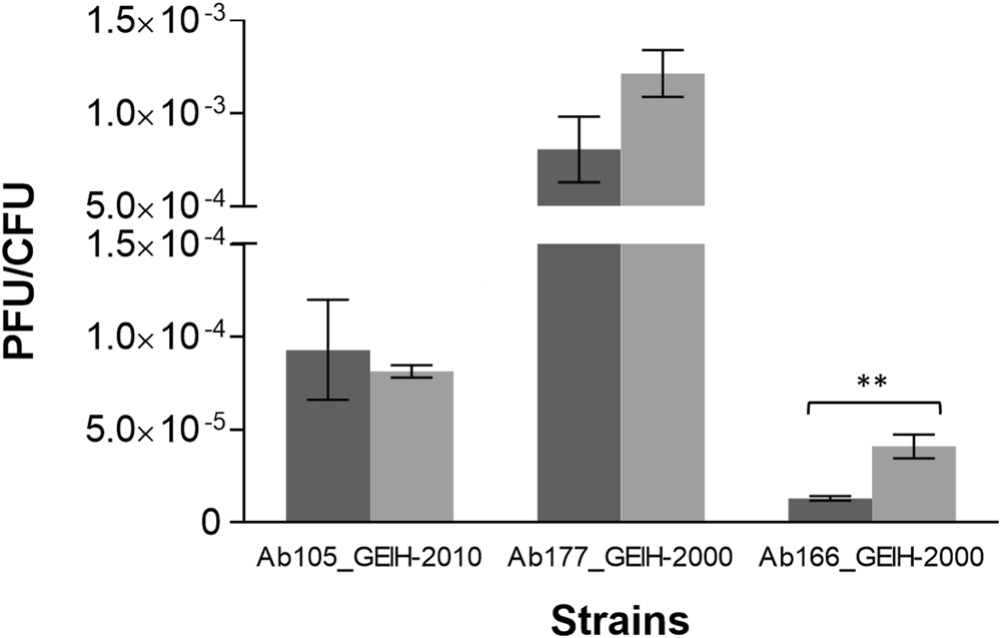
Effect of the QS/QQ network (carrying the AidA enzyme) on the bacteriophage infection capacity. Three strains of *A*. *baumannii* (Ab105_GEIH-2010, Ab177_GEIH-2000 and Ab166_GEIH-2000) were infected with Ab105-1φ and Ab105-2φ, both in the presence and absence of the 3-oxo-C12-HSL molecule (light grey and dark grey, respectively), used as a QS inhibitor in *A*. *baumanni*. **Indicates statistically significant differences (two-tailed Student t-test and Welch’s t-test, P < 0.05).

The Ab_GEIH-2010 strains, all of which carried a functional network of QS/QQ mechanisms (including the AidA protein), were infected with the bacteriophages (Ab105-1φ and Ab105- 2φ). In all Ab_GEIH-2010 isolates, as well as in strain Ab177_GEIH-2000 (which harboured a functional QS/QQ network including the AidA protein), the infective capacity (PFU/CFU) of the bacteriophages (Ab105-1φ and Ab105- 2φ) was not statistically significant different in the presence or absence of the 3-oxo-C12-HSL molecule. However, the bacteriophages were not able to infect those Ab_GEIH-2000 strains lacking the AidA protein (Ab175_GEIH-2000 and Ab192_GEIH-2000) or that displayed low expression thereof (Ab158_GEIH-2000 and Ab183_GEIH-2000), or those strains with mutations or deletions on the AbaR protein (Ab161_GEIH-2000 and Ab169_GEIH-2000) (Table 4). Moreover, a statistically significant increase in the infective capacity of the bacteriophages (Ab105-1φ and Ab105-2φ) was found in the only strain in the study that did not possess AidA, AbaI or AbaR proteins (Ab166_GEIH-2000), in the presence of the external 3-oxo-C12-HSL molecule (Fig. 7).

## Discussion

Viruses that infect bacteria (bacteriophages) can influence the dynamics of the bacterial community, the evolution of the bacterial genome and the biogeochemistry of the ecosystem. However, the degree of influence differs depending on whether the bacteriophages establish lytic, chronic or lysogenic infections. The analysis of different ecosystems by comprehensive modelling will help provide a better understanding of the diverse lifestyles and ecological impacts of lysogens in nature^9,20^. In this study of the genomic evolution in 18 *A*. *baumannii* strains belonging to ST-2 clone isolated in the same ICU in 2000 (9 strains referred to collectively as Ab_GEIH-2000) and in 2010 (9 strains referred to collectively as Ab_GEIH-2010), we found that the Ab_GEIH-2010 strains harboured two conserved temperate bacteriophages (Ab105-1ϕ and Ab105-2ϕ), which displayed lytic activity and activated the SOS response (in the presence of MMC). Microarray assays revealed overexpression of three viral proteins associated with protection of the virus against bacterial attack during the lytic activation of the bacteriophages. Moreover, qRT-PCR studies confirmed the overexpression of the genes coding the three proteins, 40 min after induction with MMC: i) the SAM-dependent methyltransferases or AdoMet-MTases (ORF27 from Ab 105-2ϕ), which have been associated with protection of the viral genome of the host restriction enzymes from the bacteria^21,22^; and ii) MazG (ORF06), which is a pyrophosphohydrolase enzyme located in bacteriophages that infect *Burkholderia cenocepacia* and in marine bacteriophages (especially cyanophages), thus facilitating viral infection in the environment^23^. In *Escherichia coli*, the MazG protein has been associated with a decrease in activity of the MazF toxin (MazEF toxin-antitoxin system), a defence mechanism that inhibits the spread of phage P1^24^; and iii) the mutation-inducing UmuC protein may have significant implications for the evolution of virulence and antibiotic resistance^25,26^.

Several are the studies where has been demonstrated by NGS analysis the high degree of variation in *A*. *baumannii* clinical isolates, including single nucleotide polymorphisms (SNPs) and large DNA fragment variations in the resistance island (RI) regions, the type VI secretion system (T6SS) and the bacteriophages^27,28^. One of the key determinants of the size, composition, structure and development of a microbial community is the predation pressure exerted by bacteriophages^29^. Bacteria have accordingly evolved a battery of anti-phage defence strategies^30,31^. Evidence that QS signalling may be involved in regulating the response to phages is increasing^32^. For example, in *Escherichia coli* in the presence of QS signals, i.e. N-acyl-L-homoserine lactones (AHLs), there is a significant reduction in levels of the phage receptor LamB^33^, which protects the bacterium against attack from the λ phage. Moreover, in *Vibrio anguillarum*, mutants that are permanently locked in a high-cell density state are almost completely immune to the phage KVP40, due to the QS-mediated downregulation of the OmpK receptor used by the phage^34–36^. In other pathogens such as *Pseudomonas aeruginosa*, the QS system has also been associated with populations evolving with bacteriophages^37,38^. It has recently been demonstrated that the presence of lysogenic bacteriophages acts as a powerful driving force for the selection of functional bacterial QS systems both *in vitro* and *in vivo* in *Pseudomonas aeruginosa* strains, by stabilizing bacterial cooperation and therefore virulence^38^. Interestingly, in all strains of the Ab_GEIH-2010 collection, a new QQ enzyme (AidA) and the AbaI protein were overexpressed in the presence of exogenous 3-oxo-C12-HSL (a QS inhibitor) and H_2_O_2_ (ROS response), respectively. These results indicated the presence of a functional QS/QQ network in these cells^13^. These molecular features were not present in the Ab_GEIH-2000 strains (lacking or displaying low expression of the AidA protein as well as mutations or deletions in AbaR protein), hence showing a QS/QQ-deficient^38^, which was not infected by the Ab105-1ϕ and Ab105-2ϕ bacteriophages.

Interestingly, in a single strain which did not possess AidA, AbaI or AbaR, the low infective capacity of the bacteriophages (Ab105-1φ and Ab105-2φ) increased significantly (**P < 0.05, Student’s t-Test) in the presence of the external 3-oxo-C 12 -HSL molecule. The AidA enzyme was previously described by our research group in clinical strains of *A*. *baumannii* (non surface motile strains) associated with bacterial competition, as it is capable of hydrolyzing the signalling molecules (including 3-oxo-C12-HSL) mediated between species^13^. Weiland and colleagues described several QQ enzymes displaying hydrolytic activity against AHLs and AI-2 signals^39,40^. Therefore, several authors propose the use, together with lytic phage therapy, of QS modulators, i.e. quorum quenchers, to decrease the phage resistance mediated by the QS system^38,41,42^.

Another molecular difference between the strains in the two collections (Ab_GEIH-2000 and Ab_GEIH-2010) was the presence/absence of a plasmid harbouring genes encoding the OXA 24/40 ß-lactamase (resistance to carbapenems) and AbkA/AbkB proteins. This AbkA/AbkB toxin-antitoxin (TA) system was described by Mosqueda *et al*. in 2014^19^ in all plasmids carrying the *bla*_OXA 24/40_ ß-lactamase gene that confers resistance to carbapenems in strains of *A*. *baumannii* in Spanish hospitals. In plasmids, TA systems have been associated with plasmid stabilization^43,44^, although it has been hypothesised that TA loci serve only to maintain plasmid DNA at the expense of the host organism^45^. Other authors suggest that these systems have evolved to favour the competitive ability of plasmids in cell progeny^46,47^. This hypothesis has been corroborated by the results of computer modelling^46^. The clinical antibiotic-resistant *Acinetobacter baumannii* strains have been shown to display higher susceptibility to environmental phages than antibiotic-sensitive strains^48^. Our results regarding the clinical multiresistant strains in the Ab_GEIH-2010 collection, which showed higher sensitivity to the Ab105-1ϕ and Ab105-2ϕ phages, is also consistent with the aforementioned finding.

In conclusion, two main molecular changes occurred during adaptation of clinical *A*. *baumannii* strains to a hospital environment during a decade: i) acquisition of a plasmid harbouring genes for OXA 24/40 ß-lactamase (associated with resistance to carbapenems) and AbKA/AbkB proteins (TA system), implicated in plasmid stabilization, and ii) acquisition of two temperate bacteriophages, Ab105-1ϕ (63 proteins) and Ab105-2ϕ (93 proteins), containing important proteins associated with protection of the viral genome against bacterial attack (the SAM-dependent methyltransferases or AdoMet-MTases, as well as MazG and UmuC). Interestingly, the Ab_GEIH-2000 strains showed a QS/QQ-deficient network relative to the functional QS/QQ network observed in Ab_GEIH-2010 strains under stress conditions, which could indicate the molecular evolution to a functional network of QS and QQ cells by these temperate bacteriophages (Ab105-1ϕ and Ab105-2ϕ) in the Ab_GEIH-2010 collection. The functional QS/QQ network included a new QQ enzyme, AidA, which acts as a bacterial defence mechanism and was overexpressed in the presence of a QS inhibitor (exogenous 3-oxo-C12-HSL). The aforementioned molecular characteristics have an important influence on the evolution of bacterial pathogens and how these adapt to the host environment.

## Methods

### Isolates of *A*.*baumannii* from Ab_GEIH-2000/2010 collections

We studied eighteen genetically related clinical strains of A. *baumannii* (ST2 indicated by Multilocus Sequence Typing, MLST) isolated from patients in the same ICU of a Spanish hospital, in 2000 (Ab_GEIH-2000 group of strains) and 2010 (Ab_GEIH-2010 group of strains)^49,50^ (Umbrella BioProject PRJNA422585 Acc.Number in NCBI server). The strains were identified during the II Spanish Multicentre Study (GEIH-REIPI *Acinetobacter baumannii* 2000-2010) in which 654 strains of *A*. *baumannii* were isolated in 42 participating hospitals^50,51^.

We determined the antibiotic susceptibility profile by microdilution, according to CLSI recommendations^50^. We used PCR to determine the presence of the OXA 24/40 ß-lactamase and an AbkA/AbkB toxin-antitoxin system in all strains from the Ab_GEIH-2000 and Ab_GEIH-2010 collections.

### Analysis of the genomes from Ab_GEIH-2000/2010 collections by next generation sequencing (NGS)

The Ab_GEIH-2000 and Ab_GEIH-2010 strains (including Ab105_GEIH-2010 and Ab155_GEIH-2000 isolates) were analyzed by next generation sequencing (NGS) in a Roche 454 GS FLX+ sequencer and Illumina MiSeq system. Isolates Ab155_GEIH-2000 and Ab105_GEIH-2010 reads were assembled using newbler Roche assembler. The other isolates were assembled using Velvet (Velvet v1.2.10 (https://www.ebi.ac.uk/~zerbino/velvet/). Putative ORFs were predicted from assembled contigs using the GeneMarkS gene prediction program^52^, which was previously trained with the *Acinetobacter baumannii* genome (GI:83207914). Blast2Go^53^ and RAST^54^ were used for functional annotation of each predicted protein. rRNA and tRNA were identified using RNAmmer^55^ and tRNAscan-SE 1.21^56^.

The phylogenetic tree with multiple alignment was developed by Progressive Mauve software (http://www.darlinglab.org/mauve/mauve.html) and painted through dendroscope tool (http://www.dendroscope.org/).

Construction of the pan genomes (all proteins), core genomes (similar proteins) and accessory genomes (no similar proteins) of the Ab_GEIH-2000 and Ab_GEIH-2010 strains was carried out using the PanSeq.^57^ and Spine tools^58^.

The bacteriophage sequence was isolated from Ab105_GEIH-2010 (a strain representative of the Ab_GEIH-2010 collection) and manually assembled to improve the continuity of phage sequences. PCR amplification was used to confirm *in silico* assembly results used as a negative control for the Ab155_GEIH-2000 strain. Reconstructed phage tools^59,60^. All phage proteins detected were manually annotated using the Protein BLAST^61^ and InterProScan tools^62^ and displayed ≥50% protein homology. The *in silico* assembly results were confirmed by PCR amplification.

The bacteriophage genomes of all strains analyzed in this study were compared following the indications of Krzywinski and collaborators^63^.

### Study of the temperate bacteriophages of Ab105_GEIH-2010 (representative strain from Ab_GEIH-2010 collection)

Mitomycin C (MMC)-induced bacteriophages were analyzed in the Ab105_ GEIH-2010 strains by three methods: (i) gene expression by microarrays and qRT-PCR; (ii) growth curves; and (iii) isolation of the bacteriophages and transmission electron microscopy (TEM) studies.

#### Gene expression studies by microarrays and qRT-PCR

For the microarray studies, we obtained Dnase-treated RNA from a mid-exponential growth phase culture (optical density at 600 nm, 0.5). The cultures were treated with MMC (at a final concentration of 10 μg ml^−1^) and incubated for 1 h to induce an SOS response. The samples were removed for RNA extraction with the High Pure RNA Isolation Kit (Roche, Germany). The corresponding controls were processed in the same way but without addition of the above-mentioned compounds.

The microarrays were specifically designed for the Ab 105_GEIH-2010 isolates by Bioarray Diagnostico Genético (Alicante, Spain) and using eArray (Agilent). The microarray assays were performed with 15,744 probes to study 4,017 genes. Labelling was carried out by two-colour microarray-based prokaryote analysis and Fair Play III labelling, version 1.3 (Agilent). Three independent RNAs per condition (biological replicates) were used in each experiment. Statistical analysis was carried out using Bioconductor, implemented in the RankProd software package for the R computing environment. A gene was considered induced when the ratio of the treated to the untreated preparation was ≥1.5 and the P value was <0.05^13^.

Quantitative real-time PCR (qRT-PCR) was used to examine the expression of bacteriophage genes in relation to interaction with the bacterial host (host-virus interactions). We used the Lightcycler 480 RNA MasterHydrolysis Probe (Roche, Germany) for the qRT-PCR studies. The UPL Taqman Probes (Universal Probe Library-Roche, Germany), Taqman probes and primers used are listed in Table S1 (Supplementary files). We adjusted the concentrations of the samples to achieve efficiencies of 90–110% and performed all experiments in triplicate from three RNA extractions (50 ng per RNA sample). For each strain, we normalized the expression of all genes relative to the single-copy *rpoB* housekeeping gene. We then calibrated the normalized expression of each gene of interest relative to its expression by untreated Ab_GEIHs RNA, which was assigned a value of 1.0.

#### Growth curves

About 100 ml LB broth was inoculated with 1:100 (v/v) of clinical strain Ab105_GEIH-2010 and was cultured overnight under shaking at 37 °C. In order to induce bacteriophages, one of the bacterial cultures, in which the optical density (OD) at 600 nm had reached 0.6, was exposed to MMC (Fischer Scientific, Loughborough, UK) at a final concentration of 10 µg/ml. Aliquots (1 ml) of the culture were removed for RNA extraction and the OD was measured every 20 minutes, and then every hour.

#### Isolation of the bacteriophages and TEM studies

Broth culture of strain Ab105_GEIH-2010 was induced as previously described for bacteriophage induction. Lysates were centrifuged at 3400 × g for 10 min and the supernatant was filtered through a 0.22 nm filter (Millipore). NaCl was added, to a final concentration of 0.5 M, and the suspensions were mixed thoroughly and left on ice for 1 h. The suspensions were then centrifuged at 3400 × g for 40 min at 4 °C, and the supernatants were transferred to sterile tubes. PEG 6000 (10% wt/vol) was added and dissolved by rocking the tubes at room temperature for 1 h and subsequent overnight incubation at 4 °C. Bacteriophages were then precipitated at 3400 × g for 40 min at 4 °C and resuspended in SM buffer (0.1 M NaCl, 1 mM MgSO_4_, 0.2 M Tris-HCl, pH 7.5)^64^. The samples were stored at 4 °C until being processed for TEM. The samples were negatively stained with 1% aqueous uranyl acetate before being examined in a JEOL JEM-1011 electron microscope.

### QS/QQ network in Ab_GEIH-2000/2010 collections: QS/QQ genes

We used qRT-PCR to examine expression of the network of QS/QQ genes (the *abaI* gene from the QS system and the *aidA* gene from the QQ system)^13^. We obtained DNAse-treated RNA from cultures treated with either non-native 3-oxo-C12-HSL (associated with inhibition of the QS system)^13,14^ or H_2_O_2_ (ROS response implicated in activation of the QS system)^13,65^. We used the Lightcycler 480 RNA MasterHydrolysis Probe (Roche, Germany) for the qRT-PCR studies. The UPL Taqman Probes (Universal Probe Library-Roche, Germany), Taqman probes and primers used are listed in Table S1 (Supplementary files). We adjusted the concentrations of the samples to yield efficiencies of 90–110% and normalized them with housekeeping gene (*rpoB*), as previously mentioned.

We also studied the surface motility (Quorum Sensing phenotype)^13^ of all strains. The motility assays were performed in plates of modified LB-LN (nutrient depleted)^66^ containing 2 g tryptone, 1 g yeast extract and 5 g NaCl. Assays were carried out with 0.25% Difco (Bacto^TM^ agar)^13^.

### Extraction of bacteriophages of Ab105_GEIH-2010 (representative strain from Ab_GEIH-2010 collection)

The lysogenic bacteriophages (Ab105-1φ and Ab105-2φ) were isolated from a culture of *A*. *baumannii* strain Ab105_GEIH-2010, obtained by culturing the bacterium in LB medium at 37 °C until it reached the late log phase of growth. In order to lyse the cells and obtain the bacteriophages contained within, the culture was first incubated in the presence of 10% chloroform for 30 minutes and centrifuged at 3000 × g for 15 minutes before the supernatant was recovered and filtered through Millipore 0.22 μm membranes. The bacteriophages were then concentrated by the double agar overlay method^67^ and used to infect *A*. *baumannii* strain Ab177_GEIH-2000, which did not harbour any bacteriophages. The plates were washed with 3 ml of phage buffer (10 mM Tris-HCL pH 7.5 and 1.8 MgSO 4) under stirring for 3 hours, after which the buffer was recovered and the phages were refiltered.

### Estimation of bacterial sensitivity to bacteriophages from Ab_GEIH-2000/2010 collections

All strains from the Ab_GEIH-2000/2010 collections were grown overnight. The cultures were subsequently diluted 1:100 in LB medium and in LB medium supplemented with 10 μM 3-oxo-C12-HSL (exogenous AHL that inhibits the detection of quorum in *A*. *baumannii*). When the OD at 600 nm reached 0.1, the cultures were infected with the previously purified bacteriophages of strain Ab105_GEIH-2010 at a multiplicity of infection (MOI) of 10 (calculated by dividing the number of bacteria [colony-forming units CFUs] the number of phages [plaque-forming units, PFUs], in a given volume of infection mixture). The cultures were then incubated at 37 °C and shaken at 180 rpm for 4 hours. An aliquot of each culture was used to establish the number of CFUs by means of serial dilution. The bacteriophages were then extracted as described above and finally the PFUs were enumerated by the double layer agar method using strain Ab177_GEIH-2000 as substrate for infection.

### Nucleotide sequence accession number

The WGS and arrays studies of the Ab_GEIH-2000 and Ab_GEIH-2010 strain collections form part of the II Spanish Multicentre Study. GEIH-REIPI *Acinetobacter baumannii* 2000-2010 project (Umbrella BioProject PRJNA422585).

## Electronic supplementary material

Supplementary Information

## References

[1] del Mar Tomas M (2005). Hospital outbreak caused by a carbapenem-resistant strain of *Acinetobacter baumanni*i: patient prognosis and risk-factors for colonisation and infection. Clin Microbiol Infect.

[2] Garnacho-Montero J (2016). *Acinetobacter baumannii* in critically ill patients: Molecular epidemiology, clinical features and predictors of mortality. Enferm Infecc Microbiol Clin.

[3] Fournier PE, Richet H (2006). The epidemiology and control of *Acinetobacter baumannii* in health care facilities. Clin Infect Dis.

[4] Diene SM (2013). The rhizome of the multidrug-resistant *Enterobacter aerogene*s genome reveals how new “killer bugs” are created because of a sympatric lifestyle. Mol Biol Evol.

[5] Dijkshoorn L, Nemec A, Seifert H (2007). An increasing threat in hospitals: multidrug-resistant *Acinetobacter baumannii*. Nat Rev Microbiol.

[6] Obeng N, Pratama AA, Elsas JD (2016). The Significance of Mutualistic Phages for Bacterial Ecology and Evolution. Trends Microbiol.

[7] Lynch KH, Stothard P, Dennis JJ (2010). Genomic analysis and relatedness of P2-like phages of the *Burkholderia cepacia* complex. BMC Genomics.

[8] Matos, R. C. *et al*. *Enterococcus faecalis* prophage dynamics and contributions to pathogenic traits. *PLoS Genet* **9**(6) 10.1371/journal.pgen.1003539 (2013). 10.1371/journal.pgen.1003539 PMC3675006 23754962

[9] Howard-Varona C, Hargreaves KR, Abedon ST, Sullivan MB (2017). Lysogeny in nature: mechanisms, impact and ecology of temperate phages. ISME J.

[10] Dong YH, Wang LY, Zhang LH (2007). Quorum-quenching microbial infections: mechanisms and implications. Philos Trans R Soc Lond B Biol Sci.

[11] Ochiai S, Yasumoto S, Morohoshi T, Ikeda T (2014). AmiE, a novel N-acylhomoserine lactone acylase belonging to the amidase family, from the activated-sludge isolate A*cinetobacter* sp. strain Ooi24. Appl Environ Microbiol.

[12] Bzdrenga J (2017). Biotechnological applications of quorum quenching enzymes. Chem Biol Interact..

[13] López, M. *et al*. GEIH-GEMARA (SEIMC). Quorum sensing network in clinical strains of *A*. *baumanni*i: AidA is a new quorum quenching enzyme. *PLoS One* **22**;12(3), 10.1371/journal.pone.0174454 (2017). 10.1371/journal.pone.0174454 PMC5362224 28328989

[14] Stacy DM, Welsh MA, Rather PN, Blackwell HE (2012). Attenuation of quorum sensing in the pathogen *Acinetobacter baumannii* using non-native N-Acyl homoserine lactones. ACS Chem Biol.

[15] Ghigo JM (2001). Natural conjugative plasmids induce bacterial biofilm development. Nature.

[16] Dougherty, K. *et al*. Multiple phenotypic changes associated with large-scale horizontal gene transfer. *PLoS One* **21**(9(7)), 10.1371 (2014). 10.1371/journal.pone.0102170 PMC4105467 25048697

[17] Zgoda A (2001). A relationship between RP4 plasmid acquisition and phenotypic changes in *Pseudomonas fluorescens* R2fN. Antonie Van Leeuwenhoek.

[18] Harrison, E. *et al*. Plasmid carriage can limit bacteria-phage coevolution. *Biol Lett* **11**(8), 10.1098/rsbl.2015.0361 (2015). 10.1098/rsbl.2015.0361 PMC4571675 26268992

[19] Mosqueda N (2014). GEIH-GEMARA (SEIMC) and REIPI. Characterization of plasmids carrying the *bla*_OXA-24/40_ carbapenemase gene and the genes encoding the AbkA/AbkB proteins of a toxin/antitoxin system. J Antimicrob Chemother.

[20] Alivisatos AP (2015). Unified Microbiome Initiative Consortium.MICROBIOME. A unified initiative to harness Earth’s microbiomes. Science.

[21] Murphy J, Mahony J, Ainsworth S, Nauta A, van Sinderen D (2013). Bacteriophage orphan DNA methyltransferases: insights from their bacterial origin, function, and occurrence. Appl Environ Microbiol.

[22] Murphy J (2014). Methyltransferases acquired by lactococcal 936-type phage provide protection against restriction endonuclease activity. BMC Genomics.

[23] Lynch KH, Stothard P, Dennis JJ (2012). Comparative analysis of two phenotypically-similar but genomically-distinct *Burkholderia cenocepacia*-specific bacteriophages. BMC Genomics.

[24] Hazan R, Engelberg-Kulka H (2004). *Escherichia coli* mazEF-mediated cell death as a defense mechanism that inhibits the spread of phage P1. Mol Genet Genomics.

[25] Hare JM, Ferrell JC, Witkowski TA, Grice AN (2014). Prophage induction and differential RecA and UmuDAb transcriptome regulation in the DNA damage responses of *Acinetobacter baumannii* and *Acinetobacter baylyi*. PLoS One.

[26] Touchon M (2014). The genomic diversification of the whole *Acinetobacter* genus: origins, mechanisms, and consequences. Genome Biol Evol.

[27] Liu F (2014). Comparative genomic analysis of *Acinetobacter baumannii* clinical isolates reveals extensive genomic variation and diverse antibiotic resistance determinants. BMC Genomics..

[28] Graña-Miraglia L (2017). Rapid Gene Turnover as a Significant Source of Genetic Variation in a Recently Seeded Population of a Healthcare-Associated Pathogen. Front Microbiol..

[29] Chibani-Chennoufi S, Bruttin A, Dillmann ML, Brüssow H (2004). Phage-host interaction: an ecological perspective. J Bacteriol.

[30] Miller MB, Bassler BL (2001). Quorum sensing in bacteria. Annu Rev Microbiol.

[31] Labrie SJ, Samson JE, Moineau S (2010). Bacteriophage resistance mechanisms. Nat Rev Microbiol.

[32] Hyman P, Abedon ST (2010). Bacteriophage host range and bacterial resistance. Adv Appl Microbiol.

[33] Høyland-Kroghsbo NM, Maerkedahl RB, Svenningsen SL (2013). A quorum-sensing-induced bacteriophage defense mechanism. MBio.

[34] Tan D, Gram L, Middelboe M (2014). Vibriophages and their interactions with the fish pathogen *Vibrio anguillarum*. Appl Environ Microbiol.

[35] Tan D, Dahl A, Middelboe M (2015). Vibriophages Differentially Influence Biofilm Formation by *Vibrio anguillarum* Strains. Appl Environ Microbiol.

[36] Diggle SP, Cornelis P, Williams P, Cámara M (2006). 4-quinolone signalling in *Pseudomonas aeruginosa*: old molecules, new perspectives. Int J Med Microbiol.

[37] Schuster M, Sexton DJ, Diggle SP, Greenberg EP (2013). Acyl-homoserine lactone quorum sensing: from evolution to application. Annu Rev Microbiol.

[38] Saucedo-Mora MA (2017). Selection of Functional Quorum Sensing Systems by Lysogenic Bacteriophages in *Pseudomonas aeruginosa*. Front Microbiol.

[39] Weiland-Bräuer N, Pinnow N, Schmitz RA (2015). Novel reporter for identification of interference with acyl homoserine lactone and autoinducer-2 quorum sensing. Appl Environ Microbiol.

[40] Weiland-Bräuer N, Kisch MJ, Pinnow N, Liese A, Schmitz RA (2016). Highly Effective Inhibition of Biofilm Formation by the First Metagenome-Derived AI-2 Quenching Enzyme. Front Microbiol.

[41] Hoque MM (2016). Quorum Regulated Resistance of *Vibrio cholerae* against Environmental Bacteriophages. Sci Rep.

[42] Moreau P, Diggle SP, Friman VP (2017). Bacterial cell-to-cell signaling promotes the evolution of resistance to parasitic bacteriophages. Ecol Evol.

[43] Jurenaite M, Markuckas A, Suziedeliene E (2013). Identification and characterization of type II toxin-antitoxin systems in the opportunistic pathogen *Acinetobacter baumannii*. J Bacteriol.

[44] Fernández-García, L. *et al*. Toxin-Antitoxin Systems in Clinical Pathogens. *Toxins* (*Basel*) **20**;8(7), 10.3390/toxins8070227 (2016) 10.3390/toxins8070227 PMC4963858 27447671

[45] Van Melderen L, Saavedra De Bast M (2009). Bacterial toxin-antitoxin systems: more than selfish entities?. PLoS Genet.

[46] Mochizuki A, Yahara K, Kobayashi I, Iwasa Y (2006). Genetic addiction: selfish gene’s strategy for symbiosis in the genome. Genetics.

[47] Van Melderen L (2010). Toxin-antitoxin systems: why so many, what for?. Curr Opin Microbiol.

[48] Chen LK (2017). Clinical Antibiotic-resistant *Acinetobacter baumannii* Strains with Higher Susceptibility to Environmental Phages than Antibiotic-sensitive Strains. Sci Rep.

[49] Villar M (2014). GEIH/GEMARA/REIPI-Ab20101 Group. Epidemiologic and clinical impact of *Acinetobacter baumannii* colonization and infection: a reappraisal. Medicine (Baltimore).

[50] Fernández-Cuenca F (2013). grupo del proyecto GEIH-REIPI-Ab 2010. *In vitro* activity of 18 antimicrobial agents against clinical isolates of *Acinetobacter* spp.: multicenter national study GEIH-REIPI-Ab 2010. Enferm Infecc Microbiol Clin.

[51] Rumbo C (2013). Spanish Group of Nosocomial Infections and Mechanisms of Action and Resistance to Antimicrobials (GEIH-GEMARA); Spanish Society of Clinical Microbiology and Infectious Diseases (SEIMC); Spanish Network for Research in Infectious Diseases(REIPI). Contribution of efflux pumps, porins, and β-lactamases to multidrug resistance in clinical isolates of *Acinetobacter baumannii*. Antimicrob Agents Chemother.

[52] Lukashin AV, Borodovsky M (1998). GeneMark.hmm: new solutions for gene finding. Nucleic Acids Res.

[53] Conesa A (2005). Blast2GO: a universal tool for annotation, visualization and analysis in functional genomics research. Bioinformatics.

[54] Aziz RK (2008). The RAST Server: rapid annotations using subsystems technology. BMC Genomics.

[55] Lagesen K (2007). RNAmmer: consistent and rapid annotation of ribosomal RNA genes. Nucleic Acids Res.

[56] Lowe TM, Eddy SR (1997). tRNAscan-SE: a program for improved detection of transfer RNA genes in genomic sequence. Nucleic Acids Res.

[57] Laing C (2010). 2010. Pan-genome sequence analysis using Panseq: an online tool for the rapid analysis of core and accessory genomic regions. BMC Bioinformatics.

[58] Ozer EA, Allen JP, Hauser AR (2014). Characterization of the core and accessory genomes of *Pseudomonas aeruginosa* using bioinformatic tools Spine and AGEnt. BMC Genomics.

[59] Zhou Y, Liang Y, Lynch KH, Dennis JJ, Wishart DS (2011). PHAST: a fast phage search tool. Nucleic Acids Res.

[60] Arndt D (2016). PHASTER: a better, faster version of the PHAST phage search tool. Nucleic Acids Res..

[61] Kent WJ (2002). 2012. BLAT-the BLAST-like alignment tool. Genome Res.

[62] Zdobnov EM, Apweiler R (2001). InterProScan–an integration platform for the signature-recognition methods in InterPro. Bioinformatics.

[63] Krzywinski M (2009). Circos: an information aesthetic for comparative genomics. Genome Res..

[64] Hargreaves KR, Colvin HV, Patel KV, Clokie JJ, Clokie MR (2013). Genetically diverse *Clostridium difficile* strains harboring abundant prophages in an estuarine environment. Appl Environ Microbiol.

[65] Bhargava N, Sharma P, Capalash N (2014). Pyocyanin stimulates quorum sensing-mediated tolerance to oxidative stress and increases persister cell populations in *Acinetobacter baumannii*. Infect Immun.

[66] Clemmer KM, Bonomo RA, Rather PN (2011). Genetic analysis of surface motility in *Acinetobacter baumannii*. Microbiology..

[67] Kropinski AM (2009). Measurement of the rate of attachment of bacteriophage to cells. Methods Mol Biol.

