# Supplementary material for: Evolution of the Quorum network and the mobilome (plasmids and bacteriophages) in clinical strains of Acinetobacter baumannii during a decade
Source: Sci Rep. 2018 Feb 6;8:2523. doi: 10.1038/s41598-018-20847-7 (PMC5802823; doi:10.1038/s41598-018-20847-7)
Supplement: Supplementary file 1 — Supplementary Information [file 41598_2018_20847_MOESM1_ESM.pdf]

# SUPPLEMENTARY INFORMATION

## «Evolution of the Quorum network and the mobilome (plasmids and bacteriophages) in clinical strains of *Acinetobacter baumannii* during a decade»

M. López, A. Rueda , J.P. Florido, L. Blasco, L. Fernández-García, R. Trastoy, F. Fernández-Cuenca, L. Martínez-Martínez, J. Vila, A. Pascual, G. Bou and M. Tomás

**Table 1S**. (Supplementary files). Primers and probes used in the qRT-PCR studies. Taqman Probe UPL, Universal Probe Library (Roche, Germany).

**Table S2.** (Supplementary files). Genome and functional description of proteins (ORFs) in the Ab 105-1ϕ prophage. ORFs: Open Reading Frames from GenMark software. HP/UF, Hypothetical Protein/Unknown Function.

**Table S3.** (Supplementary files). Genome and functional description of proteins (ORFs) in the Ab 105-2ϕ prophage. ORFs: Open Reading Frames from GenMark software. HP/UF, Hypothetical Protein/Unknown Function.

| PRIMERS and PROBES for qRT-PCR STUDIES | | | | |
|---|---|---|---|---|
| **Bacterial Interaction (Ab105-2ϕ prophage)** | | **Primer Sequence (5´-3´)** | **Taqman Probe (UPL)** | **References** |
| *SAM-methyltransferase or adoMet-MTase* (ORF27) | Fow<br>Rev | GGTGATGTTCGTGATGTGTTG<br>CAACGATTGCCAGCTACAGA | 88/CATCCTCC | This study |
| *mazG-like* (ORF06) | Fow<br>Rev | ATAAGGCACGAATTCATCAGG<br>GATTTTCGCTGCGTGGTTAG | 136/TGATGAGC | This study |
| *umuC* (ORF93) | Fow<br>Rev | TCGGCGGAAGTATGTATCAA<br>TTTTTAGCATACCCTCAAGAGTAAAAT | 116/AGCCTGGT | This study |
| **Quorum Sensing** | | **Primer Sequence (5´-3´)** | **Taqman Probes (UPL)** | **References** |
| *abaI* | Fow<br>Rev | CCGCTACAGGGTATTTGTTGAAT<br>GCAGGGAATAGGCATTCCATTG | 6FAM-<br>TGGATTCTCTGTCTTGAGCCACGACA-<br>BBQ | This study |
| **Quorum Quenching** | | **Primer Sequence (5´-3´)** | **Taqman Probes** | **References** |
| *aidA* | Fow<br>Rev | GGGAACTTCTTTCGGTGGAG<br>AACAGCAGCAAGTCGATTATCA | 145/CAGCCACC | This study |
| | Fow<br>Rev | GGGACTTCTTTCGGTGGAG<br>GCAGCAAGCCGGTTATCA | 145/ CAGCCACC | This study |
| **Housekeeping** | | **Primer Sequence (5´-3´)** | **Taqman Probes** | **References** |
| *rpoB* | Fow<br>Rev | CGTGTATCTGCGCTTGG<br>CGTACTTCGAAGCCTGCAC | 131/CTGGTGGT | (Carlos et al., 2013) |

**Table 1S.**

| ORF | Start | End | Strand | Length | Protein Blast (Coverage ≥50%) | PHASTER database |
|---|---|---|---|---|---|---|
| ORF01 | 1 | 1263 | + | 1263 | Integrase protein | PHAGE_Pseudo_PMG1_NC_016765:integrase;phage (gi374531680) |
| ORF02 | 1269 | 1538 | - | 270 | Helix-turn-helix domain protein | PHAGE_Acinet_Bphi_B1251_NC_019541: HP; phage (gi423262035) |
| ORF03 | 1539 | 1796 | - | 258 | Molybdopterin oxidoreductase | HP |
| ORF04 | 1800 | 2084 | - | 285 | - | HP |
| ORF05 | 2081 | 2290 | - | 210 | - | HP |
| ORF06 | 2281 | 2538 | - | 258 | - | PHAGE_Acinet_Bphi_B1251_NC_019541: HP; phage (gi423262038) |
| ORF07 | 2540 | 3541 | - | 1002 | - | PHAGE_Acinet_Bphi_B1251_NC_019541: HP; phage (gi423262039) |
| ORF08 | 3538 | 4659 | - | 1122 | AAA+ ATPases-domain protein | PHAGE_Acinet_Bphi_B1251_NC_019541: phage nucleotide-binding protein (gi423262040) |
| ORF09 | 4671 | 4994 | - | 324 | - | PHAGE_Acinet_Bphi_B1251_NC_019541: HP; phage (gi423262041) |
| ORF10 | 4997 | 5437 | - | 441 | - | HP |
| ORF11 | 5635 | 6390 | - | 756 | Cro/CI family transcriptional repressor | PHAGE_Pseudo_vB_PaeP_Tr60_Ab31_NC_023575: Cro/CI transcriptional regulator; phage (gi589286922) |
| ORF12 | 6491 | 6706 | + | 216 | Cro/CI family transcriptional repressor | PHAGE_Escher_HK75_NC_019541: regulatory protein Cro; phage (gi356870715) |
| ORF13 | 6717 | 7073 | + | 357 | Cro/CI family transcriptional repressor | PHAGE_Acinet_Bphi_B1251_NC_019541: HP; phage (gi423261985) |
| ORF14 | 7136 | 7408 | + | 273 | - | PHAGE_Acinet_Bphi_B1251_NC_019541: HP; phage (gi423261986) |
| ORF15 | 7405 | 7701 | + | 297 | - | PHAGE_Acinet_Bphi_B1251_NC_019541: HP; phage (gi423261987) |
| ORF16 | 7698 | 8054 | + | 357 | - | PHAGE_Acinet_Bphi_B1251_NC_019541: HP; phage (gi423261988) |
| ORF17 | 8054 | 8983 | + | 930 | - | PHAGE_Thalas_BA3_NC_009990: HP; phage (gi160700630) |
| ORF18 | 8976 | 9725 | + | 750 | - | PHAGE_Erwini_PEp14_NC_016767:HP; phage (gi374531902) |
| ORF19 | 9722 | 10144 | + | 423 | - | PHAGE_Acinet_Bphi_B1251_NC_019541:HP; phage (gi423261991) |
| ORF20 | 10134 | 10556 | + | 423 | - | PHAGE_Acinet_LZ35_NC_031117: exonuclease; phage (gi100082) |
| ORF21 | 10549 | 10773 | + | 225 | - | PHAGE_Acinet_Bphi_B1251_NC_019541: HP; phage (gi423261992) |
| ORF22 | 10773 | 11168 | + | 396 | Endonuclease-like family | PHAGE_Acinet_vB_AbaS_TRS1_NC_031098: holing; phage (gi100062) |
| ORF23 | 11165 | 11665 | + | 501 | - | HP |
| ORF24 | 11960 | 12511 | + | 552 | - | HP |
| ORF25 | 12773 | 13531 | + | 759 | - | HP |
| ORF26 | 13626 | 14273 | + | 648 | Transposase | PHAGE_Pectob_ZF40_NC_019522: transposase; phage (gi422936679) |
| ORF27 | 14321 | 14830 | + | 510 | - | PHAGE_Pectob_ZF40_NC_019522: HP; phage (gi422936680) |
| ORF28 | 14827 | 16485 | + | 1659 | Terminase TerL | PHAGE_Salmon_SEN34_NC028699: Terminase; phage (gi966201419) |
| ORF29 | 16496 | 17908 | + | 1413 | HI1409 family phage-associated protein | PHAGE_Salmon_SEN34_NC_028699: Portal protein; phage (gi966201420) |
| ORF30 | 17931 | 18566 | + | 636 | Head morphogenesis protein | PHAGE_Salmon_SEN34_NC_028699: Head morphogenesis (gi966201421) |

| | | | | | | |
|---|---|---|---|---|---|---|
| ORF31 | 18628 | 18714 | + | 87 | - | HP |
| ORF32 | 18855 | 20171 | + | 1317 | Putative Head protein/prohead protease | PHAGE_Salmon_SEN34_NC_028699: HP; phage (gi966201422) |
| ORF33 | 20175 | 20651 | + | 477 | - | PHAGE_Salmon_SEN34_NC_028699: HP; phage (gi966201423) |
| ORF34 | 20716 | 21741 | + | 1026 | Phage capside | PHAGE_Salmon_SEN34_NC_028699: HP; phage (gi966201424) |
| ORF35 | 21751 | 22182 | + | 432 | Polynucleotide phosphorylase/polyadenylase | HP |
| ORF36 | 22186 | 22572 | + | 387 | - | PHAGE_Salmon_SEN34_NC_028699: HP; phage (gi966201426) |
| ORF37 | 22569 | 23129 | + | 561 | - | PHAGE_Salmon_SEN34_NC_028699: HP; phage (gi966201427) |
| ORF38 | 23116 | 23484 | + | 369 | Head-tail adaptor | PHAGE_Salmon_SEN34_NC_028699: HP; phage (gi966201428) |
| ORF39 | 23487 | 24026 | + | 540 | - | PHAGE_Salmon_SEN34_NC_028699: HP; phage (gi966201429) |
| ORF40 | 24030 | 25505 | + | 1476 | - | PHAGE_Salmon_SEN34_NC_028699: HP; phage (gi966201430) |
| ORF41 | 25520 | 25963 | + | 444 | - | PHAGE_Salmon_SEN34_NC_028699: HP; phage (gi966201431) |
| ORF42 | 25963 | 26424 | + | 462 | - | PHAGE_Salmon_SEN34_NC_028699: HP; phage (gi966201432) |
| ORF43 | 26620 | 28644 | + | 2025 | Tail tape measure protein | PHAGE_Salmon_SEN34_NC_028699: Tail tape measure; phage (gi966201433) |
| ORF44 | 28641 | 28997 | - | 357 | - | HP |
| ORF45 | 28997 | 29224 | - | 228 | - | HP |
| ORF46 | 29229 | 29417 | + | 189 | - | HP |
| ORF47 | 29584 | 30180 | + | 597 | - | PHAGE_Salmon_SEN34_NC_028699: HP; phage (gi966201434) |
| ORF48 | 30183 | 30479 | + | 297 | - | PHAGE_Salmon_SEN34_NC_028699: HP; phage (gi966201435) |
| ORF49 | 30476 | 31435 | + | 960 | - | PHAGE_Salmon_SEN34_NC_028699: HP; phage (gi966201436) |
| ORF50 | 31438 | 32100 | + | 663 | Baseplate assembly protein J-like | PHAGE_Salmon_SEN34_NC_028699: Baseplate assembly protein; phage (gi966201439) |
| ORF51 | 32135 | 32488 | + | 354 | - | PHAGE_Salmon_SEN34_NC_028699: HP; phage (gi966201441) |
| ORF52 | 32491 | 33675 | + | 1185 | Baseplate assembly protein J-like | PHAGE_Salmon_SEN34_NC_028699: HP; phage (gi966201442) |
| ORF53 | 33675 | 34265 | + | 591 | - | PHAGE_Psychr_pOW2O_A_NC_020841: HP; phage (gi472339849) |
| ORF54 | 34258 | 34866 | + | 609 | Tail-collar fiber family protein | PHAGE_Aeromo_25_NC_008208: gp36 small distal tail fiber; phage (gi109290197) |
| ORF55 | 34930 | 36984 | + | 2055 | Tail fiber protein | PHAGE_Acinet_IME_200_NC_028987: Tail fiber protein; phage (gi971763488) |
| ORF56 | 36986 | 37213 | + | 228 | - | HP |
| ORF57 | 37291 | 37680 | + | 390 | - | PHAGE_Acinet_Bphi_B1251_NC_019541: HP; phage (gi423262030) |
| ORF58 | 37723 | 38268 | + | 546 | Glycosyl hydrolase family | PHAGE_Acinet_Bphi_B1251_NC_019541: HP; phage (gi423262031) |
| ORF59 | 38458 | 39138 | + | 681 | - | PHAGE_Acinet_Bphi_B1251_NC_019541: HP; phage (gi423262032) |
| ORF60 | 39149 | 39796 | + | 648 | - | PHAGE_Acinet_Bphi_B1251_NC_019541: HP; phage (gi423262033) |
| ORF61 | 39803 | 40396 | + | 594 | - | PHAGE_Acinet_Bphi_B1251_NC_019541: HP; phage (gi423262034) |

| | | | | | | |
|---|---|---|---|---|---|---|
| ORF62 | 40401 | 40550 | - | 150 | DNA polymerase UmuC | HP |
| ORF63 | 40642 | 41496 | - | 855 | Transposase | PROPHAGE_Escher_CFT073: Transposase insF; phage (gi26249410) |

**Table 2S.**

| ORF | Start | End | Strand | Length | Protein Blast (Coverage ≥50%) | PHASTER database |
|---|---|---|---|---|---|---|
| ORF01 | 190 | 1326 | - | 1137 | Integrase | PHAGE_Entero_HK022_NC_002166: integrase; phage(gi9634144) |
| ORF02 | 1331 | 1594 | - | 264 | - | - |
| ORF03 | 1658 | 2140 | - | 483 | Methyltransferase (SAM or AdoMet-MTase) | PHAGE_Gordon_Nymphadora_NC_031061: DNA polymerase III subunit epsilon; phage(gi100067) |
| ORF04 | 2137 | 2346 | - | 210 | - | - |
| ORF05 | 2343 | 2876 | - | 534 | Phage protein | - |
| ORF06 | 2873 | 3208 | - | 336 | MazG-like protein | PHAGE_Citrob_Moon_NC_027331: HP; phage(gi849248535) |
| ORF07 | 3205 | 3516 | - | 312 | - | PHAGE_Roseob_1_NC_015466: HP RDJLphi1_gp43;phage(gi331028097) |
| ORF08 | 3516 | 3779 | - | 264 | - | - |
| ORF09 | 3772 | 4896 | - | 1125 | AAA+ ATPases-domain protein | PHAGE_Acinet_Bphi_B1251_NC_019541: putative phage nucleotide-binding protein; phage(gi423262040) |
| ORF10 | 4908 | 5231 | - | 324 | - | PHAGE_Acinet_Bphi_B1251_NC_019541: HP; phage(gi423262041) |
| ORF11 | 5234 | 5674 | - | 441 | - | - |
| ORF12 | 5903 | 6145 | + | 243 | - | - |
| ORF13 | 6152 | 6355 | - | 204 | - | - |
| ORF14 | 6667 | 7059 | - | 393 | Putative membrane protein | - |
| ORF15 | 7062 | 8063 | - | 1002 | - | PHAGE_Shigel_SfIV_NC_022749: HP; phage(gi557307575) |
| ORF16 | 8114 | 8329 | - | 216 | - | PHAGE_Acinet_Bphi_B1251_NC_019541: HP; phage(gi423261982) |
| ORF17 | 8350 | 9024 | - | 675 | Lex A-like protein | PHAGE_Burkho_BcepC6B_NC_005887: putative prophage repressor protein; phage(gi48697234) |
| ORF18 | 9126 | 9326 | + | 201 | XRE family transcriptional regulator (repressor) | - |
| ORF19 | 9337 | 9699 | + | 363 | Transcriptional regulator | PHAGE_Acinet_Bphi_B1251_NC_019541: HP; phage(gi423261985) |
| ORF20 | 9901 | 10074 | + | 174 | - | - |
| ORF21 | 10071 | 10847 | + | 777 | Helix-turn-helix domain-containing protein | PHAGE_Acinet_LZ35_NC_031117: HP; phage(gi100053) |
| ORF22 | 10847 | 12172 | + | 1326 | Replicative DNA helicase | PHAGE_Acinet_AP22_NC_017984: putative replicative DNA helicase; phage(gi388570844) |
| ORF23 | 12169 | 12393 | + | 225 | - | - |
| ORF24 | 12390 | 12617 | + | 228 | Integrase | - |
| ORF25 | 12614 | 12865 | + | 252 | DnaB-like helicase C terminal domain protein | - |
| ORF26 | 12862 | 13038 | + | 177 | - | - |
| ORF27 | 13035 | 13691 | + | 657 | Methyltransferase (SAM or AdoMet-MTase) | PHAGE_Cellul_phi18:1_NC_021790: HP; phage(gi526177063) |
| ORF28 | 13688 | 14011 | + | 324 | - | PHAGE_Acinet_LZ35_NC_031117: nucleoside triphosphate hydrolase; phage(gi100081) |
| ORF29 | 14011 | 14208 | + | 198 | - | - |

| | | | | | | |
|---|---|---|---|---|---|---|
| ORF30 | 14219 | 14392 | + | 174 | DnaJ-class molecular chaperone with C-terminal Zn finger domain, putative | PHAGE_Acinet_AP22_NC_017984: DnaJ-class molecular chaperone with C-terminal Zn finger domain, putative; phage(gi388570792) |
| ORF31 | 14394 | 14807 | + | 414 | - | PHAGE_Acinet_vB_AbaS_TRS1_NC_031098: hemolysin XhlA; phage(gi100061) |
| ORF32 | 14804 | 15256 | + | 453 | - | PHAGE_Marino_P12026_NC_018269: HP; phage(gi399528341) |
| ORF33 | 15256 | 15336 | + | 81 | - | - |
| ORF34 | 15345 | 15821 | + | 477 | - | - |
| ORF35 | 15882 | 16211 | + | 330 | - | - |
| ORF36 | 16345 | 16731 | + | 387 | - | - |
| ORF37 | 16744 | 17097 | + | 354 | - | - |
| ORF38 | 17148 | 17615 | + | 468 | - | PHAGE_Acinet_Bphi_B1251_NC_019541: HP; phage(gi423261997) |
| ORF39 | 17584 | 18225 | + | 642 | - | PHAGE_Acinet_Bphi_B1251_NC_019541: HP;; phage(gi423261998) |
| ORF40 | 18285 | 18842 | + | 558 | - | PHAGE_Acinet_Bphi_B1251_NC_019541: phage protein; phage(gi423261999) |
| ORF41 | 18826 | 20142 | + | 1317 | Terminase-like family | PHAGE_Pseudo_YMC11/02/R656_NC_028657: HP; phage(gi966197881) |
| ORF42 | 20182 | 21528 | + | 1347 | - | PHAGE_Acinet_Bphi_B1251_NC_019541: HP; phage(gi423262001) |
| ORF43 | 21538 | 22644 | + | 1107 | Phage head morphogenesis | PHAGE_Acinet_Bphi_B1251_NC_019541: phage putative head morphogenesis protein; phage(gi423262002) |
| ORF44 | 22803 | 23081 | + | 279 | - | PHAGE_Acinet_Bphi_B1251_NC_019541: HP; phage(gi423262003) |
| ORF45 | 23180 | 23422 | + | 243 | - | PHAGE_Acinet_Bphi_B1251_NC_019541: HP; phage(gi423262004) |
| ORF46 | 23639 | 23830 | + | 192 | - | PHAGE_Acinet_Bphi_B1251_NC_019541: HP; phage(gi423262005) |
| ORF47 | 23938 | 24693 | + | 756 | - | PHAGE_Caulob_rogue_NC_019408: HP; phage(gi414088930) |
| ORF48 | 24707 | 25657 | + | 951 | Methyltransferase | PHAGE_Entero_phiFL3A_NC_013648: coat protein; phage(gi281416261) |
| ORF49 | 25702 | 26037 | + | 336 | HeH-LEM domain protein | PHAGE_Acinet_Bphi_B1251_NC_019541: HP; phage(gi423262008) |
| ORF50 | 26041 | 26421 | + | 381 | Periplasmic binding protein | PHAGE_Acinet_Bphi_B1251_NC_019541: HP; phage(gi423262009) |
| ORF51 | 26422 | 26790 | + | 369 | Glutamate 5-kinase | PHAGE_Acinet_Bphi_B1251_NC_019541: HP; phage(gi423262010) |
| ORF52 | 26799 | 27203 | + | 405 | - | PHAGE_Acinet_Bphi_B1251_NC_019541: HP; phage(gi423262011) |
| ORF53 | 27217 | 27543 | + | 327 | - | PHAGE_Acinet_Bphi_B1251_NC_019541: HP; phage(gi423262012) |
| ORF54 | 27545 | 27943 | + | 399 | - | PHAGE_Acinet_Bphi_B1251_NC_019541: HP; phage(gi423262013) |
| ORF55 | 27945 | 28163 | + | 219 | - | PHAGE_Acinet_Bphi_B1251_NC_019541: HP; phage(gi423262014) |
| ORF56 | 28257 | 28610 | + | 354 | DNA-binding transcriptional activator MhpR | HAGE_Acinet_Bphi_B1251_NC_019541: HP; phage(gi423262015) |
| ORF57 | 28610 | 29707 | + | 1098 | Phage tail protein | - |
| ORF58 | 29617 | 33480 | - | 3864 | Tail length tape-measure protein 1 | PHAGE_Acinet_Bphi_B1251_NC_019541: tail tape measure protein; phage(gi423262023) |

| | | | | | | |
|---|---|---|---|---|---|---|
| ORF59 | 33541 | 33888 | - | 348 | - | - |
| ORF60 | 34035 | 34550 | - | 516 | Rha family phage transcriptional regulator | PHAGE_Acinet_vB_AbaS_TRS1_NC_031098: replication protein DnaD; phage(gi100014) |
| ORF61 | 34968 | 35906 | - | 939 | BRO family, N-terminal domain protein | PHAGE_Endosy_APSE_1_NC_000935: P43; phage(gi9633590) |
| ORF62 | 36049 | 36429 | + | 381 | - | - |
| ORF63 | 36446 | 37369 | - | 924 | - | - |
| ORF64 | 37433 | 37957 | - | 525 | - | PHAGE_Flavob_1H_NC_031911: HP; phage(gi100008) |
| ORF65 | 38062 | 38520 | - | 459 | Transcriptional regulator | - |
| ORF66 | 38529 | 38828 | - | 300 | - | - |
| ORF67 | 39330 | 39845 | - | 516 | - | PHAGE_Acinet_Bphi_B1251_NC_019541: HP; phage(gi423262018) |
| ORF68 | 39915 | 40832 | - | 918 | - | PHAGE_Acinet_Bphi_B1251_NC_019541: HP; phage(gi423262017) |
| ORF69 | 40885 | 43965 | - | 3081 | Bacteriophage protein | PHAGE_Acinet_Bphi_B1251_NC_019541: HP; phage(gi423262029) |
| ORF70 | 43958 | 44320 | - | 363 | - | PHAGE_Acinet_Bphi_B1251_NC_019541: HP; phage(gi423262028) |
| ORF71 | 44317 | 44823 | - | 507 | - | PHAGE_Acinet_Bphi_B1251_NC_019541: HP; phage(gi423262027) |
| ORF72 | 44823 | 45221 | - | 399 | - | PHAGE_Acinet_Bphi_B1251_NC_019541: HP; phage(gi423262026) |
| ORF73 | 45271 | 45762 | - | 492 | Phage tail tape measure protein | PHAGE_Acinet_Bphi_B1251_NC_019541: tail tape measure protein; phage(gi423262023) |
| ORF74 | 45762 | 46814 | - | 1053 | Phage tail tape measure protein | PHAGE_Acinet_Bphi_B1251_NC_019541: tail tape measure protein; phage(gi423262023) |
| ORF75 | 46894 | 49326 | - | 2433 | Tail length tape-measure protein 1 | PHAGE_Acinet_Bphi_B1251_NC_019541: tail tape measure protein; phage(gi423262023) |
| ORF76 | 49388 | 50329 | - | 942 | - | - |
| ORF77 | 50381 | 51133 | - | 753 | Phage antirepressor BRO family | PHAGE_Salmon_SPN3UB_NC_019545: putative antirepressor family protein; phage(gi423262399) |
| ORF78 | 51130 | 51516 | - | 387 | - | - |
| ORF79 | 51596 | 51820 | - | 225 | DNA-binding protein | PHAGE_Salmon_vB_SemP_Emek_NC_018275: transcriptional repressor protein; phage(gi399498811) |
| ORF80 | 51880 | 52146 | + | 267 | Regulatory protein mnt | PHAGE_Pseudo_phi3_NC_030940: HP; phage(gi100009) |
| ORF81 | 52175 | 52315 | - | 141 | - | - |
| ORF82 | 52693 | 53208 | - | 516 | - | PHAGE_Acinet_Bphi_B1251_NC_019541: HP; phage(gi423262018) |
| ORF83 | 53278 | 54195 | - | 918 | - | PHAGE_Acinet_Bphi_B1251_NC_019541: HP; phage(gi423262017) |
| ORF84 | 54249 | 54536 | - | 288 | Phage tail protein | PHAGE_Acinet_Bphi_B1251_NC_019541: putative tail fiber; phage(gi423262016) |
| ORF85 | 54665 | 55294 | + | 630 | Bacteriophage protein | PHAGE_Acinet_Bphi_B1251_NC_019541: HP; phage(gi423262029) |

| | | | | | | |
|---|---|---|---|---|---|---|
| ORF86 | 55363 | 55752 | + | 390 | - | PHAGE_Acinet_Bphi_B1251_NC_019541: HP; phage(gi423262030) |
| ORF87 | 55951 | 56271 | + | 321 | Extracellular lipase, Pla-1/cef family | PHAGE_Pseudo_YMC11/02/R656_NC_028657: HP; phage(gi966197843) |
| ORF88 | 56374 | 57117 | + | 744 | - | PHAGE_Acinet_Bphi_B1251_NC_019541: HP; phage(gi423262021) |
| ORF89 | 57131 | 57415 | - | 285 | - | - |
| ORF90 | 57557 | 58504 | + | 948 | Alpha/beta hydrolase (Bro-N family) | PHAGE_Endosy_APSE_1_NC_000935: P43; phage(gi9633590) |
| ORF91 | 58633 | 58782 | + | 150 | Putative membrane protein | - |
| ORF92 | 58843 | 59385 | + | 543 | Glycosyl hydrolase 108 | PHAGE_Acinet_Bphi_B1251_NC_019541: HP; phage(gi423262031) |
| ORF93 | 59621 | 60814 | - | 1194 | Lesion bypass DNA polymerase V (UmuC) | PHAGE_Salmon_vB_SosS_Oslo_NC_018279: error-prone lesion bypass DNA polymerase V; phage(gi399528790) |

**Table 3S.**
